# Pharmacovigilance Signals of the Opioid Epidemic over 10 Years: Data Mining Methods in the Analysis of Pharmacovigilance Datasets Collecting Adverse Drug Reactions (ADRs) Reported to EudraVigilance (EV) and the FDA Adverse Event Reporting System (FAERS)

**DOI:** 10.3390/ph15060675

**Published:** 2022-05-27

**Authors:** Stefania Chiappini, Rachel Vickers-Smith, Amira Guirguis, John M. Corkery, Giovanni Martinotti, Daniel R. Harris, Fabrizio Schifano

**Affiliations:** 1 Psychopharmacology, Drug Misuse and Novel Psychoactive Substances Research Unit, School of Life and Medical Sciences, University of Hertfordshire, Hertfordshire AL10 9EU, UK; stefaniachiappini9@gmail.com (S.C.); j.corkery@herts.ac.uk (J.M.C.); giovanni.martinotti@gmail.com (G.M.); f.schifano@herts.ac.uk (F.S.); 2 Department of Epidemiology, University of Kentucky College of Public Health, 111 Washington Avenue, Lexington, KY 40536, USA; 3 Department of Pharmacy, Swansea University Medical School, The Grove, Swansea University, Swansea, Wales SA2 8PP, UK; amira.guirguis@swansea.ac.uk; 4 Department of Neurosciences, Imaging and Clinical Sciences, Università degli Studi G. D’Annunzio, 66100 Chieti-Pescara, Italy; 5 Institute for Pharmaceutical Outcomes and Policy, University of Kentucky College of Pharmacy, 289 South Limestone Street, Lexington, KY 40536, USA; daniel.harris@uky.edu; 6 Center for Clinical and Translational Sciences, University of Kentucky, 800 Rose Street, Lexington, KY 40506, USA

**Keywords:** opioids, prescription drug abuse, drug misuse, drug dependence, adverse events, pharmacovigilance, new psychoactive substances

## Abstract

In the past twenty years, the consumption of opioid medications has reached significant proportions, leading to a rise in drug misuse and abuse and increased opioid dependence and related fatalities. Thus, the purpose of this study was to determine whether there are pharmacovigilance signals of abuse, misuse, and dependence and their nature for the following prescription opioids: codeine, dihydrocodeine, fentanyl, oxycodone, pentazocine, and tramadol. Both the pharmacovigilance datasets EudraVigilance (EV) and the FDA Adverse Events Reporting System (FAERS) were analyzed to identify and describe possible misuse-/abuse-/dependence-related issues. A descriptive analysis of the selected Adverse Drug Reactions (ADRs) was performed, and pharmacovigilance signal measures (i.e., reporting odds ratio, proportional reporting ratio, information component, and empirical Bayesian geometric mean) were computed for preferred terms (PTs) of abuse, misuse, dependence, and withdrawal, as well as PTs eventually related to them (e.g., aggression). From 2003 to 2018, there was an increase in ADR reports for the selected opioids in both datasets. Overall, 16,506 and 130,293 individual ADRs for the selected opioids were submitted to EV and FAERS, respectively. Compared with other opioids, abuse concerns were mostly recorded in relation to fentanyl and oxycodone, while tramadol and oxycodone were more strongly associated with drug dependence and withdrawal. Benzodiazepines, antidepressants, other opioids, antihistamines, recreational drugs (e.g., cocaine and alcohol), and several new psychoactive substances, including mitragynine and cathinones, were the most commonly reported concomitant drugs. ADRs reports in pharmacovigilance databases confirmed the availability of data on the abuse and dependence of prescription opioids and should be considered a resource for monitoring and preventing such issues. Psychiatrists and clinicians prescribing opioids should be aware of their misuse and dependence liability and effects that may accompany their use, especially together with concomitant drugs.

## 1. Introduction

### 1.1. The Opioid Epidemic

In the past decades, abuse of medications has been increasingly reported, including several prescription medicines (e.g., quetiapine, pregabalin, gabapentin, etc.) and over-the-counter (OTC) medicines (e.g., loperamide, dextromethorphan, promethazine, etc.) [1,2,3,4], but mostly noted in relation to the non-medical use of pharmaceutical opioids [5]. The so-called opioid epidemic registered in the United States (US) was characterized by cyclical waves of heroin use and the non-medical use of pharmaceutical opioids, and their consequent fatalities [5]. Those opioids included opiates, which are naturally occurring alkaloids found in the opium poppy, such as morphine and codeine; its semi-synthetic derivatives, e.g., heroin, hydrocodone, and oxycodone; and a range of synthetic or pharmaceutical opioids, such as methadone, tramadol, and fentanyl [6]. More recently, however, there has been an alarming rate of opioid overdose deaths due to illicitly manufactured fentanyl, fentanyl analogues [5,7,8,9,10], and other chemicals, known as ‘research opioids’ or novel synthetic opioids (NOSs),which are molecules initially developed by pharmaceutical companies with the aim of producing more effective opioids for pain management, but later discarded or discontinued during phase III clinical trials due to unwanted adverse effects, and considered unsuitable for further development [11]. Due to their potency, many of those substances, e.g., U-47700, and some fentanyl analogues, such as butyrylfentanyl, have been put under international control in recent years [11,12].

Doubling the estimated number of past-year opioid users worldwide, in 2019, opioid users (including both people using opiates and people using pharmaceutical opioids for non-medical purposes) were estimated to be 62 million, corresponding to 1.2% of the global population aged 15–64 [5]. Over the same period, the prevalence of opioid use increased by 76%, whereas the global population increased by 10% [5]. The opioid epidemic followed an increased availability of prescription opioids, possibly derived from the rise of efficient global supply chains, the liberalization of laws governing opioids’ prescriptions, lax prescribing practices, and the ability to purchase online from illicit pharmacies, the greatest increase being recorded with oxycodone [10,13,14]. Furthermore, issues identified in the US following the opioid epidemic were high rates of neonatal opioid-withdrawal syndrome, poor socioeconomic conditions, rising concomitant use of heroin and fentanyl, outbreaks of injection-related infectious diseases, and relatively higher rates of unemployment in areas with high opioid prescribing [5,10].

In Europe, the medical use and non-medical use of prescription opioids have also increased, but this increase was modest compared with the US [11,13,15]. Data from a task force of the European Pain Federation, including 25 European countries, considering trends of opioid-related harms over the last 20 years, recorded an increase in the number of opioid prescriptions from 2004 to 2016, especially in France, Finland, and the Netherlands, while, in the United Kingdom (UK) there was a rise in opiate/opioid overdose deaths between 2016 and 2018, but not in opioid prescriptions [16]. Despite this, and with the exception of the UK, fatalities related to the misuse of prescription opioids have hardly been reported in Europe [17,18]. Moreover, in an analysis of trends in prescription opioid use and opioid-related issues from 2010 to 2018 in 19 European countries, there was a general increase in prescription opioid consumption; the largest increase and the highest consumption was recorded in the UK, as compared to the rest of EU. According to the same study, in 2018, among all included European countries, Scotland showed the highest rate of high-risk opioid users, opioid-related hospital admissions, opioid-related deaths, opioid-use-disorder treatment admissions, and opioid-substitution-therapy patients. Countries with high rates of opioid-related issues were Northern Ireland (synthetic and other opioids), Ireland (heroin and methadone), and England (all opioids) [19]. Consistently, poisonings involving opioids in the UK have been increasing dramatically since 2014; deaths related to tramadol rose by 240 per annum during the past twenty years, mirroring tramadol-related prescribing levels [20]. Other significant events were the introduction of fentanyl analogues into the illegal drug market through online sales [5] and the replacement of heroin by fentanyl and buprenorphine in Estonia and Finland, respectively, thereby establishing these drugs on the opioid market [13]. Similar to data regarding opioid misuse, numbers regarding opioid-related deaths are currently at much lower levels than in the US; however, they still represent a large preventable health burden [16,17,18].

### 1.2. Post-Marketing Studies

Post-marketing evidence regarding opioid abuse, misuse, and dependence is limited. Data from the Research Abuse, Diversion, and Addiction-Related Surveillance (RADARS^®^) System Programs have been used to analyze rates of abuse, misuse, and diversion of both methadone and buprenorphine in the US, finding a steady increase for both the molecules from 2003 to 2007; the rate ratios of abuse, misuse, and diversion were consistently higher for methadone than buprenorphine, and the numbers of exposures requiring medical attention corresponded to 46.8% and 25.8% of all cases, respectively. Interestingly, the most commonly diverted form (73%) of methadone was solid oral tablets, which are typically dispensed at pharmacies, not at opioid treatment programs [21]. The analysis of fentanyl-related misuse, abuse, dependence, and withdrawal cases reported from 2004 to 2018 to pharmacovigilance systems, such as the European Medicines Agency (EMA), the Yellow Card Scheme (YCS), and the Food and Drug Administration (FDA) Adverse Event Reporting System (FAERS), showed increasing levels over time, with drug dependence/withdrawal, intentional product misuse/drug abuse, and overdose as the most commonly reported adverse events, and a significant number of these requiring prolonged hospitalization or resulting in death [7]. Using the FAERS dataset to determine the frequency counts of fifteen different opioid drugs, we can see that oxycodone, hydrocodone, and fentanyl accounted for more than half of the reports, with the highest frequency count for oxycodone; the opioid with the highest proportion of deaths to drug count was heroin (71.8%), followed by dextromethorphan (55.6%), methadone (37.2%), and morphine (26.8%) [22]. Finally, there are several published studies regarding the use of pharmaceutical opioid-abuse-deterrent formulations and their effectiveness [23,24,25,26].

*Aim of the study*: The present study aimed at analyzing two pharmacovigilance datasets, the EudraVigilance (EV) and the FAERS, in order to describe how abuse, misuse, dependence, and withdrawal issues have been recorded for several opioids, i.e., codeine, dihydrocodeine, fentanyl, oxycodone, pentazocine, and tramadol, and detect possible signals of disproportionality. A descriptive analysis of the adverse drug reactions (ADRs) recorded, including anagraphic characteristics, country of origin, most common diagnoses, concomitant licit/illicit drugs, and routes of administration of the selected opioids, was performed. Finally, the new psychoactive substances (NPS), considered as new narcotic or psychotropic drugs, in pure form or in preparation, that are not controlled by the United Nations drug conventions, but which may pose a public health threat comparable to that posed by substances listed in these conventions [11], such as synthetic cannabinoids, synthetic cathinones, new benzodiazepines, and synthetic opioids [8,11,27], have been described when involved.

## 2. Results

### 2.1. EMA versus FAERS Datasets

**Figure 1:**
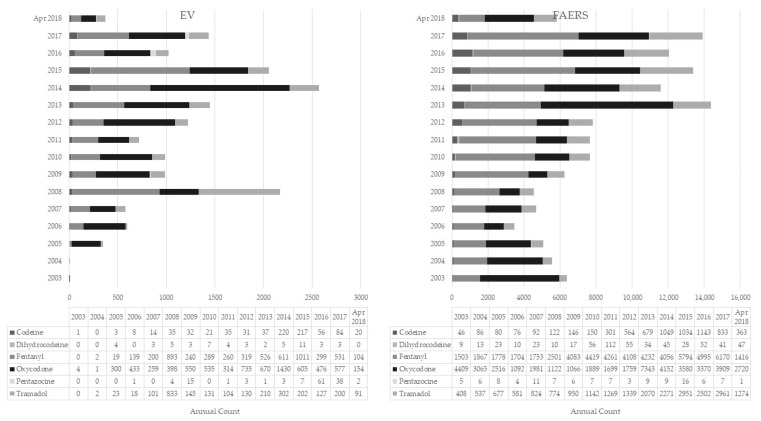
Number of cases involving opioids recorded in the European Medicines Agency (EMA) and Food and Drug Administration (FDA) Adverse Event Reporting System databases.

From February 2003 to April 2018, a total of 16,506 and 130,293 ADR reports involving the selected opioids were submitted to EV and FAERS, respectively. Among these EV reports, 45% involved oxycodone (*n* = 7441), 33% fentanyl (*n* = 5443), 16% tramadol (*n* = 2619), 5% codeine (*n* = 814), and less than 1% each for pentazocine (*n* = 136) and dihydrocodeine (*n* = 53; Table 1). In FAERS, the largest proportion of reports involved fentanyl (42%, *n* = 54,640), followed by oxycodone (35%, *n* = 45,672) and tramadol (17%, *n* = 22,530), and the fewest reports were those involving codeine (5%, *n* = 6764), dihydrocodeine (<1%, *n* = 575), and pentazocine (<1%, *n* = 112), respectively (Table 1). There was an increase in the number of ADRs reported to the EMA and FAERS for the selected opioids during the analytic timeframe (Figure 1). In both datasets, fentanyl, oxycodone, and tramadol had the highest number of reports. In the FAERS dataset, fentanyl and tramadol had peaks in 2018, and oxycodone in 2013, while in the EV, fentanyl peaked in 2015, and oxycodone and tramadol peaked in 2014.

The mean age ranged between 37.9 and 53.2 years; in both datasets, females were mostly involved in dihydrocodeine- and tramadol-related cases, and males in oxycodone-related cases, while differences between the two datasets regarding the most represented sex existed for codeine, fentanyl, and pentazocine reports (Table 1). Most reports came from the US, except for dihydrocodeine reports, where most came from the UK in both EV and FAERS datasets, and pentazocine reports, which primarily came from Canada and Japan (Supplementary Table S1). Where specified, the most commonly recorded indications were ‘pain’ (or pain-related issues, e.g., ‘back pain’, ‘analgesic therapy’, ‘migraine’, ‘headache’, etc.), ‘drug abuse’, and ‘intentional product misuse’ (Table 1). The routes of administration included oral and intravenous, as well as more unusual routes, such as nasal/inhalation (Table 1). The most common concomitant drugs listed on ADR reports for the six selected opioids were antidepressants, benzodiazepines, other opioids, and over-the-counter (OTC) antihistamines. Concomitant recreational substances most commonly reported were cocaine and alcohol (Table 1). Fatal outcomes were recorded most often with codeine (70% of cases), fentanyl (47%), and oxycodone (31%) in the EMA dataset, and oxycodone (37%), dihydrocodeine (33%), and codeine (30%) in the FAERS (Table 1).

### 2.2. Pharmacovigilance Signals

In FAERS, misuse-/abuse-related ADRs were most often reported for oxycodone (Table 2 and Supplementary Table S2). Specifically, compared to the other selected opioids, ‘substance abuse’ was reported more than 17 times as frequently (proportional reporting ratio (PRR) = 17.61; false discovery rate (FDR) < 0.01), ‘drug abuser’ was reported more than 10 times as frequently (PRR = 10.17; FDR < 0.01), and ‘drug abuse’ more than twice as often (PRR = 2.48; FDR < 0.01) for oxycodone (Table 2 and Supplementary Table S2). Significant misuse-/abuse-related signals were also identified for fentanyl (‘drug diversion’ and ‘intentional product misuse’), codeine (‘drug abuse’ and ‘intentional product misuse’), and tramadol (‘substance use’; Table 2 and Supplementary Table S2). In EV, misuse-/abuse-related ADRs were most often reported for codeine, fentanyl, and oxycodone. Compared to the other selected opioids, ‘drug abuse’ was listed as an ADR nearly twice as frequently for codeine (PRR = 1.94), ‘drug abuser’ was recorded more than twice as often for oxycodone (PRR = 2.52), and ‘drug diversion’ more than twice as frequently for fentanyl (PRR = 2.30; all FDR < 0.01; Table 2 and Supplementary Table S2). ‘Intentional product misuse’ was reported as an ADR more than twice as frequently for codeine (PRR = 2.23) and fentanyl (PRR = 2.20), and ‘substance abuse’ was reported nearly nine times as often for oxycodone (PRR = 8.84) compared to the other opioids (all FDR < 0.01; Table 2 and Supplementary Table S2).

Conversely, dependence and withdrawal issues were primarily reported with oxycodone and tramadol, e.g., in FAERS, oxycodone and ‘drug dependence’ PRR = 11.53, ‘substance dependence’ PRR = 53.88, and ‘drug withdrawal’ PRR = 2.82; in EV, the PRR measure for tramadol and ‘dependence’ was 5.38 (all FDR < 0.01; Table 2 and Supplementary Table S2). Finally, significant signals for overdose were more commonly reported with tramadol (in EV ‘intentional overdose’ PRR = 4.00 and ‘overdose’ PRR = 1.55; all FDR < 0.01; Table 2 and Supplementary Table S2).

With regard to other PTs recorded, in all statistical measures considered and both datasets, compared with the other opioids, oxycodone was more strongly associated with ‘aggression’ and ‘euphoric mood’ (all FDR < 0.01; Supplementary Table S3). Conversely, tramadol was associated with ‘visual hallucinations’, ‘confusional state’, and ‘psychotic disorder’ (all FDR < 0.01; Supplementary Table S3).

As a secondary analysis, a pharmacovigilance analysis was conducted comparing all ADR reports for all five opioids to ADR reports for benzodiazepines (i.e., diazepam, alprazolam, clonazepam, lorazepam, delorazepam, bromazepam, flurazepam, and triazolam) in the FAERS dataset. With regard to misuse- and abuse-related ADRs, ‘drug abuse’, ‘drug diversion’, and ‘substance abuse’ were reported significantly more often for opioids than benzodiazepines (Supplementary Table S4). Dependence ADRs (i.e., ‘dependence’, ‘drug dependence’, and ‘substance dependence’) were also reported significantly more often in opioid-related reports than those containing benzodiazepines (Supplementary Table S4). ‘Drug withdrawal syndrome’ and ‘overdose’ were also more frequently reported in opioid-containing cases than benzodiazepine-containing cases (Supplementary Table S4). Lastly, ‘delirium’ and ‘euphoric mood’ were more often recorded in opioid cases when compared to benzodiazepine cases (Supplementary Table S4).

### 2.3. New Psychoactive Substances (NPS)

In both datasets, several cases recorded the abuse of NPS, such as cathinones, e.g., 4-methylethcathinone, mephedrone, methylenedioxypyrovalerone, and alpha-pyrrolidinopropiophenone (20 cases); mitragynine (15 cases); the designer benzodiazepine, flubromazolam (1 case); an unspecified phenethylamine (1 case); and the phencyclidines 3-methoxyphencyclidine and 4-methoxyphencyclidine (2 cases) (Supplementary Table S5).

## 3. Discussion

The data analyzed confirmed that diversion, abuse, and dependence are issues which might present with the selected opioids, especially if used in large or extremely large dosages, with concomitant licit/illicit drugs, and through unconventional routes of administration. In the face of a growing demand for safer drugs, our research offers a means of identifying early drug-related safety signals through large multinational datasets of ADRs. The substantial number of abuse-/dependence-related events identified provides further evidence corroborating the potential diversion of several drugs reported to be potentially misused. Although this kind of approach should only be considered as exploratory to generate signals, the disproportionality analysis in pharmacovigilance databases is a validated method in drug safety research and surveillance [28]. The post-marketing assessment of medicines plays a key role for better defining drugs safety profile in real-world setting and filling the evidence gap of pre-marketing studies, which are normally conducted on limited numbers of patients that are selected based on strict eligibility criteria and have limited duration, thus preventing the detection of rare and long-term adverse reactions [29].

### 3.1. Opioid Differences

#### 3.1.1. Epidemiology

To the best of our knowledge, this is the largest analysis of pharmacovigilance data relating to opioids misuse/abuse/dependence and withdrawal cases. Consistent with the related knowledge on this topic, current findings referred to high numbers of patients presenting with opioid misuse, abuse, and dependence issues. The study focused on different types of prescription opioids, such as codeine, dihydrocodeine, fentanyl, oxycodone, pentazocine, and tramadol. The two datasets were consistent in terms of most commonly reported opioid drugs, which were fentanyl, oxycodone, and tramadol, with differences related to the opioid with the greatest number of reports: fentanyl in the US and oxycodone in Europe. Several other factors might have influenced ADR reporting, including, for example, (i) differences in opioid drug regulation, (ii) their availability on the market, (iii) pharmaceutical advertising, (iv) prescribing attitudes of doctors, (v) level of law enforcement and governmental drug policy, (vi) regulatory frameworks for pharmaceutical drugs, and (vii) cultural reasons [17]. Moreover, numbers might have mirrored the prescribing practices of opioid medications; in Canada and the US, in the past two decades, the medical use of prescription opioids, in particular, of oxycodone, hydrocodone, and codeine, has increased up to 14-fold [17]. Furthermore, according to the 2015/2016 National Survey on Drug Use and Health, a nationally representative survey of the non-institutionalized US population, an estimated 4.4% misused non-fentanyl prescription opioids, whereas 0.1% misused prescription fentanyl, the latter of which is more commonly involved in substance-use disorders [30]. Unfortunately, studies on the use of prescription opioids in Europe are scarce and often do not distinguish prescription analgesics from OTC analgesics and prescribed from non-prescribed use [17,31]. Furthermore, the European Monitoring Centre for Drugs and Addiction (EMCDDA), which collates drug-related information in the EU, is mainly focused on heroin and synthetic opioids/fentanyl analogues than on prescription opioids [11]. According to available European data, even though not reaching the 4- to 14-fold increase identified in the US and Canada, where the use of prescription opioids is currently 2.5 to 4 times higher compared to Western Europe, the use of prescription opioids has also increased in the last decade in Europe, especially in Norway, Sweden, Finland, Netherlands, and the UK, and particularly with regard to oxycodone, fentanyl, buprenorphine, and tramadol [17]. With respect to the distribution of ADR reports by gender, women had a greater number of reports involving dihydrocodeine and tramadol in both datasets, and there was a greater number of oxycodone reports among men. These findings potentially support existing data suggesting that women are more likely to use prescription opioids compared with men, due to risk factors including depression or chronic pain conditions [32], whereas men are more likely to misuse prescription opioids primarily to feel good or get a high [33,34].

#### 3.1.2. Pharmacology

Possible reasons why fentanyl, oxycodone, and tramadol were the most recorded drugs here might be found in their pharmacological characteristics. Oxycodone is a potent semi-synthetic derivative mediating its analgesic properties through both mu opioid (MOP) receptors, which are responsible for supraspinal analgesia, respiratory depression, euphoria, sedation, decreased gastrointestinal motility, and physical dependence; and kappa opioid (KOP) receptors, which are responsible for spinal analgesia, sedation, dyspnea, dependence, dysphoria, and respiratory depression; it also has a high oral bioavailability, which can be manufactured in a time-release preparation [6]. Similar to oxycodone, in the US, fentanyl is a Schedule II substance (i.e., high potential for abuse), possessing the highest affinity for the MOP receptor [6] and the highest potency (approximately 80 times more than morphine) [9,35]. Due to these properties, fentanyl exposure in opioid-naïve individuals or those with limited opioid tolerance has been associated with significant adverse effects, such as respiratory depression and fatal overdose and, in general, to higher mortality rates than with use of shorter-acting opioid medications [30]. Codeine, dihydrocodeine, and tramadol have approximately equianalgesic potencies for oral administration, although tramadol has a different mechanism of analgesia [6]. In fact, tramadol is an atypical opioid that is thought to work through the modulation of serotonin and norepinephrine reuptake, in addition to its action as a MOP receptor agonist [6]. Although tramadol displays many of the side-effects associated with MOP receptor agonists, it is purported to produce less respiratory depression and fewer gastrointestinal side-effects than pure MOP agonists of comparable analgesic potency; thus, even when used primarily as an analgesic, it has demonstrated usefulness in treating opioid withdrawal [6,35]. Codeine, similar to oxycodone, is commonly used for chronic pain states, primarily acting on MOP receptors. Specifically, codeine, which is still available in many countries as an OTC drug, with an analgesic potency of approximately 50% of morphine and a half-life of 2.5 to 3 h, first needs to be metabolized to morphine by the body to display any activity, and, between 5% and 10% of the population is estimated to lack the ability to perform this conversion, thus deriving limited pain relief and effects [6]. In the US, codeine in its pure form is a Schedule II substance, whereas, in combination with other analgesics, it is Schedule III substance (i.e., less abuse potential than Schedule I and II substances) [35]. Similarly, dihydrocodeine is comparable in structure and in analgesic properties to codeine [35]. Different from them, pentazocine is the only member of the opioid class benzomorphans, and it is classified as a partial agonist–antagonist, having a high MOP affinity but poor MOP receptor efficacy, and thus it may act functionally as a MOP antagonist, as well as having kappa agonistic properties. Thus, used as analgesic, pentazocine has a limited effect. Moreover, psychomimetic effects (e.g., dysphoria, dysesthesias, and hallucinations) may complicate its use, particularly with increasing doses [6,35].

#### 3.1.3. Abuse and Diversion Issues

Abuse issues were most often reported in relation to fentanyl and oxycodone; fentanyl’s higher potency in comparison to the other index opioids, due to the high affinity for the MOP receptor and its strong positive reinforcing properties [7,8,36,37], make it one of the most abused, diverted, and dangerous drugs. With regard to oxycodone, in all statistical measures and both datasets, it was more strongly associated with the PTs aggression and euphoric mood compared with the other opioids considered; it is clear that euphoria might be an effect accompanying the analgesic property of opioids, and specifically mu-opioid agonists, such as oxycodone. These mood-elevating properties identified herein might be hypothetically related to the abuse issue presented above. In fact, subjective euphoric effects, unique energy, and even a sense of invincibility and relatively side-effect-free experiences have been reported by individuals that misused oxycodone [38,39,40]. Similarly, in a cross-sectional survey involving 86 patients diagnosed with opioid-dependence/opioid-use disorder and asked to answer which opioid they found the most desirable (to themselves and to their drug-using associates), which they deemed most addictive, and which served as their gateway drug to heroin, oxycodone was ranked the highest [41]. Oxycodone’s ‘likability’ and abuse and dependence liability/addictiveness has been related to its rewarding properties, linked to markedly increased active transport across the blood–brain barrier, increased phasic dopaminergic activity in the ventral–tegmental area (VTA), nucleus accumbens, and related striatal reward centers [36,41,42]. It is worth noting that the euphoric effect, higher abuse potential, and preference are described as typical of the immediate-release formulation compared to the extended-release formulation [43,44]. Conversely, increased KOP-mediated withdrawal dysphoria and other unpleasant central nervous withdrawal symptoms, such as aggressiveness, were recorded here [41]. With regard to tramadol, it appeared to be involved in both dependence and withdrawal issues (e.g., drug dependence, drug withdrawal syndrome, and substance dependence) and intentional overdose/overdose. Tramadol is a prescription opioid analgesic that is used to treat pain described as moderate to severe, post-operative pain, and off-label in restless leg syndrome in patients who have had little or no success with traditional treatments. It has also been considered for the management of withdrawal symptoms in opioid-use disorders, due to the low abuse liability and dependency risk initially perceived in comparison to other opioids [45,46]. However, following its extensive use for chronic pain relief and also in drug-abuse cases, dependency and, after long-term use, the occurrence of withdrawal symptoms were observed. Tramadol was associated with visual and auditory hallucinations, psychotic disorder, and confusional state, which might resemble the withdrawal symptoms of serotonin reuptake blockers rather than opioid blockers, and this may be related to tramadol’s mechanism of action as a serotonin and epinephrine reuptake blocker [47,48]. Consistently, a report by the Adverse Drug Reactions Advisory Committee of Australia found that confusion, hallucinations, convulsions, and serotonin syndrome were the most serious adverse reactions recorded among all tramadol-related reports [49,50]. Recently, tramadol-associated hallucinations have been proposed as a clinical entity by Jean et al. [49], speculating an involvement of several different mechanisms, such as muscarinic antagonism, serotonin reuptake inhibition, serotonin receptor-mediated dopamine dysregulation, and antagonistic effects on gamma-aminobutyric acid (GABA) receptors. Unfortunately, the data available to this study did not allow for an evaluation of the concomitant use of substances/drugs acting on the same neurotransmitter systems or organic diagnoses, and this might have influenced the clinical presentation recorded.

#### 3.1.4. Concomitant Drugs Used

Interestingly, in both the EV and FAERS datasets, the concomitant drugs most often reported with the selected opioids were benzodiazepines, antidepressants, other opioids, and OTC antihistamines; such data support the extant literature that reports that individuals misusing prescription opioids were more likely to also misuse prescription sedatives, tranquilizers, and stimulants; alcohol; and illicit drugs, e.g., cocaine [34,51,52], presenting unique problems in assessment and treatment. Considering the literature available, hypothetically, three main categories of opioid users have been identified by this study: (i) chronic users of prescription opioids who then substituted them with other opioids or decided to experiment with new opioids for recreational purposes; (ii) users of different types of opioids consecutively to self-medicate or manage withdrawal, including during opioid agonist or antagonist therapy; and (iii) opioid users inadvertently exposed to other opioids [11]. Reasons for adding other substances to opioids include enhancement of the high, compensation for undesired effects of one drug by taking another, compensation for negative internal states, or a common predisposition that is related to all substance consumption. While toxicity can be increased through pharmacokinetic or pharmacodynamic interactions and drug combinations involving opioids, specific recreational effects might be obtained through additive or synergistic rewarding effects, such as increasing dopamine release in the nucleus accumbens. In fact, preclinical studies have shown that activation of MOP receptors on gamma-amino butyric acid (GABA)-VTA cells disinhibits dopamine neurons and increases their activity and dopamine function in the nucleus accumbens; thus, even if opioid receptors are maximally occupied, a stimulant, e.g., cocaine, might increase synaptic levels of dopamine or enhance dopamine terminal release results, increasing ratings of the experienced high and desirability. Conversely, benzodiazepines often co-administered with opioids, binding GABA-A receptors resulting in the inhibition of VTA-GABA neurons, would be additive to the acute action of opioids and possibly enhancing the subjective effects of opioids, including the high, but also increasing the risk for overdose and respiratory depression [53].

#### 3.1.5. Fatalities

Regarding the outcome, the results were quite variable. Despite differences, fatal outcomes were most often reported with oxycodone and codeine in both datasets. Similar findings have been recorded in the existing literature and might possibly be influenced by several factors, including the regular use of opioids; increased opioid availability in the community or increased dosage; the use of a nervous system depressant, e.g., benzodiazepines and alcohol; injecting drug practices; and the concomitant consumption of other illicit substances, e.g., heroin, cocaine, etc. [54,55,56]. Other conditions which might have influenced the outcome are (i) past suicide attempt, (ii) presence of mental health disorders, (iii) lower levels of education, (iv) medical comorbidities, (v) middle age, and (vi) poverty [54,55,56,57]. Unfortunately, we could not understand from the present data if the opioid-involved fatalities were accidental or intentional, nor the dosage and the formulations used. Moreover, inconsistencies between datasets might be due to underreporting or missing data regarding the ADR outcome(s). Interestingly, codeine and oxycodone both exist in extended-release/controlled-release formulations, which have been marketed as abuse-deterrent formulations and have already been shown to reduce prescription opioid misuse [26,44,58]. In this respect, their introduction and increased opioid pharmacovigilance activities (e.g., updated guidelines for prescription opioids, prescription drug monitoring programs, ADR datasets such as EV and FAERS, etc.) might be considered responses to clinicians’ concerns about opioid misuse and diversion, as well as the fatalities related to prescription opioids and the opioid epidemic [59]. The increasing rates in ADR reporting over time in this study may suggest a recently growing emphasis on pharmacovigilance data [60,61,62,63], which may well provide timely, real-world, and affordable information on medication use/misuse compared to that normally recorded in controlled trials [1]. Consistent with this, prescription-based methods of drug safety surveillance might represent areas of possible progress, since combining aspects of public health surveillance, spontaneous reporting, and epidemiological studies can improve triangulation and confidence in deriving conclusions [64].

### 3.2. NPS

It is worth mentioning the presence of some NPS in the reports retrieved. One of the most represented molecules detected here was mitragynine, which has been recorded in tramadol- and oxycodone-related cases in combination with other prescription drugs (other opioids, e.g., hydromorphone and buprenorphine; benzodiazepines, e.g., alprazolam, clonazepam, and diazepam; antidepressants, e.g., mirtazapine, venlafaxine, and fluoxetine; and other drugs), the OTC loperamide, alcohol, and amphetamines (Table S5). Cathinones were the most represented NPS, including mephedrone, 4-methylethcathinone, and methylenedioxypyrovalerone. These drugs are stimulants that induce euphoria, improved psychomotor speed, alertness, and talkativeness. Acute psychiatric effects may also include dysphoria, loss of appetite, difficulty in sleeping, paranoid ideation and delusions, cognitive impairment, changes in perception, agitation, hallucinations, confusion, violence, and suicidal thoughts [65]. Interestingly, out of 20 cases involving cathinones, 10 (50%) had a fatal outcome, as is consistent with the literature available that warns of the medical toxicity issues associated with cathinones, especially if used together with other molecules; for example, cathinones might be implicated in serotonin syndrome occurrence, together with serotoninergic drugs, such as antidepressants, tramadol, etc. [65,66,67,68]. Mitragynine, found in 15 cases, is the most abundant active alkaloid in the Southeast Asian plant Mitragyna speciosa, commonly known as *kratom*. Its effects are dose-dependent: at low doses, it induces a mild stimulating effect, and at larger doses, it produces sedation and antinociception effects that are typical of opioids. Regular use may lead to dependence and opioid-like withdrawal symptoms upon discontinuation, and many related fatalities have been reported [8,69]. Unfortunately, doses for concomitant drugs were not available in this study. It would have been interesting, considering that the pharmacodynamic properties of drugs can change by using mega doses, and interactions between molecules could lead to unpredictable consequences in terms of psychotropic effects that might have justified their use, as, for example, in the case of the antidiarrhoeic drug loperamide used in supratherapeutic doses (>16 mg) to achieve euphoria (‘lope dope’) and/or avoid opioid withdrawal [70], or in the case of high doses of tramadol, inducing serotonin syndrome [71]. Interestingly, one fatal case was reported involving the abuse/overdose of tramadol, together with mitragynine; and loperamide, which presumably induced a condition of cardiotoxicity, resulting in cardiac arrest. Other NPSs reported included an unspecified phenethylamine, reported in an accidental overdose, and the designer benzodiazepine flubromazolam [63], used together with the dissociative molecules 4-Methoxyphencyclidine and 3-Methoxyphencyclidine [8], resulting in a fatal outcome. Likewise, NPSs’ safety and their toxicological and clinical profiles are still not completely understood, posing serious health risks to consumers, especially in cases of polydrug use [72].

### 3.3. Limitations

Despite the interesting findings, several limitations exist. Firstly, although the disproportionality analysis is a suitable tool to quantify signals of drug abuse, it has a limited capacity to differentiate the type of or the reason for abuse (e.g., recreational, self-medication, etc.). In addition, confounding factors such as comorbidity, dosages/routes of administration, and concomitant drugs consumed cannot be assessed properly with a pharmacovigilance approach, due to the intrinsic nature of reports used as primary sources for the study, reflecting the information as provided to EV or to the FDA by the reporter. The study of ADRs alone is rarely sufficient to confirm that a certain effect in a patient has been caused by a specific medicine, as this could have also been caused by the disease being treated, a new disease the patient developed, or by another medicine that the patient is taking. Indeed, the number of case reports for a particular medicine or suspected adverse reaction does not only depend on the real frequency of the adverse reaction but also on the extent and condition of use of the medicine, the nature of the reaction, and public awareness and compliance with reporting [73]. Thus, a single case report should be regarded only as a piece of information; further data (e.g., worldwide spontaneous case reports, clinical trials, and epidemiological studies) are needed to obtain a thorough understanding of the safety profile of an index molecule. Finally, a limitation may be related to the choice of the molecules investigated here, a choice which did not include all opioids.

## 4. Materials and Methods

Abbreviated methods are described below; detailed methods can be found in Appendix SA. The study was ethically approved in March 2018 by the University of Hertfordshire Ethics’ Committee (LMS/PGR/UH/03234).

### 4.1. Data Sources

For the present study, we requested data from the EMA [73] in April 2018 for ADR reports for the selected opioids submitted to the EV from 2003 to the present. All reports included cases where codeine, dihydrocodeine, fentanyl, oxycodone, pentazocine, and tramadol were reported as a suspected or interacting active substance. Similarly, the FAERS was queried in April 2018 for ADRs related to the selected opioids. FAERS data were available through the FAERS Public Dashboard and quarterly data extract files [74].

### 4.2. Data Analysis

We performed a descriptive analysis of ADR report characteristics, including sociodemographics, country of origin, most common diagnoses, ROA, and concomitant licit/illicit substances. IBM SPSS Statistics for Windows, version 28 (IBM Corp., Armonk, NY, USA) was used for all descriptive analyses. Pharmacovigilance signal measures, including the reporting odds ratio (ROR), proportional reporting ratio (PRR), information component (IC), and empirical Bayesian geometric mean (EBGM), were calculated in each dataset, using the R package PhViD [75]. All four pharmacovigilance measures were calculated due to differences in their sensitivity and early detection potential [76,77]; for brevity, only the PRR is shown in the text, and all calculated measures can be found in the Supplementary Materials. Given the support for the use of the false discovery rate (FDR) to identify signals over thresholds, we used an FDR < 0.05 to denote significance [78,79]. When significant signals are reported herein, all four measures meet the significance criteria. A secondary pharmacovigilance assessment of opioid ADR reports (i.e., those containing one of the five selected opioids) compared to benzodiazepine ADR reports (i.e., those containing either diazepam, alprazolam, clonazepam, lorazepam, delorazepam, bromazepam, flurazepam, triazolam, and not any of the five selected opioids) was also conducted by using FAERS data.

## 5. Conclusions

A rational and safe use of medicines incorporates the evaluation of all potential benefits and harms and their application only to indicated conditions, limiting their use to the shortest possible time and the lowest dosage in order to avoid drug toxicity in general, but also withdrawal and dependence issues. Interventions to minimize harmful patterns of prescription drug use and harm include the following: (i) a stratification assessment, including a history of legal, prescribed, and illicit drug abuse, when evaluating a new patient; (ii) abuse-deterrent formulations intended to minimize extra-medical use, e.g., making tablets tamper-resistant or including naloxone to deter injection; (iii) dedicated and multidisciplinary services for those struggling with prescribed opioid dependence and withdrawal; (iv) easy access to opioid substitution therapy and to naloxone to reduce opioid overdose deaths; and (v) promoting education on the quality use of opioids, as well as increasing awareness about opioid-related problems [3,80]. Finally, pharmacovigilance activities, aiming at the detection, assessment, understanding, and prevention of adverse effects recorded from the post-approval stage and throughout a drug’s market life, should be improved by clinicians and, in general, healthcare providers [3,81,82]. Post-marketing surveillance activities act as a supporting framework for the development of interventional strategies that will manage, prevent, and reduce the risk of ADRs in patients using medications, thereby reducing healthcare costs, e.g., regulatory actions might then include updated labeling information, restricting drug use, or product removal from the market [83].

## Figures and Tables

**Table 1:** Analysis of opioid-related adverse drug reaction reports recorded in the European Medicines Agency (EMA) EudraVigilance (EV) dataset and the Food and Drug Administration (FDA) Adverse Event Reporting System.

ADR Report Characteristics	Codeine		Dihydrocodeine		Fentanyl		Oxycodone		Pentazocine		Tramadol	
	EMA	FAERS	EMA	FAERS	EMA	FAERS	EMA	FAERS	EMA	FAERS	EMA	FAERS
Individual cases	814	6764	53	575	5443	54,640	7441	45,672	136	112	2619	22,530
Mean age in years (SD)	38.3 (13.6)	50.7 (19.6)	37.9 (12.7)	43.4 (22.2)	43.3 (16.0)	53.2 (19.2)	38.0 (13.6)	45.6 (18.2)	46.3 (16.5)	51.4 (21.1)	42.7 (15.7)	52.8 (20.4)
M (%)	73.8% (540) 26.2% (192)	32.2% (1983)	36.2% (17)	48.2% (244)	53.0% (2459)	40.5% (19,354)	61.4% (3929)	54.2% (22,504)	20.7% (28)	51.9% (54)	48.9% (1142)	38.7% (7890)
F (%)		67.8% (4167)	63.8% (30)	51.8% (262)	47.0% (2178)	59.5% (28,382)	38.6% (2468)	45.8% (19,036)	79.3% (107)	48.1% (50)	51.1% (1195)	61.3% (12,479)
Most common	Drug abuse (1.9%)	Pain (7.2%)	Pain (20.0%)	Pain (12.3%)	Pain (25.0%)	Pain (31.0%)	Drug abuse (15.3%)	Pain (30.5%)	Pain (24.4%)	Pain (17.3%)	Pain (18.9%)	Pain (21.6%)
indications recorded for the index opioid when reported (%)	Pain (1.6%)	Rheumatoid arthritis (4.9%)	Procedural pain (10.0%)	Back Pain (5.9%)	Intentional product misuse (7.3%)	Back pain (9.1%)	Pain (13.8%)	Back Pain (5.8%)	Drug abuse (7.7%)	Analgesic therapy (14.3%)	Back pain (7.8%)	Back pain (6.8%)
	Cough (1.4%)	Cough (2.6%)	Drug dependence (6.7%)	Rheumatoid arthritis (5.4%)	Back pain (4.7%)	Cancer pain (6.2%)	Back pain (4.7%)	Drug abuse (4.0%)	Migraine (3.8%)	Drug abuse (8.2%)	Headache (2.7%)	Depression (6.1%)
ROA (%)	Oral (26.9%)	Oral (32.2%)	Oral (63.0%)	Oral (40.1%)	Transdermal (44.9%)	Transdermal (75.0%)	Oral (56.0%)	Oral (76.1%)	Intravenous (70.0%)	Intramuscular (32.7%)	Oral (86.5%)	Oral (63.9%)
	Parenteral (9.0%)	Parenteral (2.3%)	Parenteral (0%)	Transplacental (16.5%)	Oral (22.6%)	Intravenous (6.0%)	Intravenous (3.2%)	Intravenous (1.3%)	Intramuscular (19.2%)	Intravenous (32.7%)	Intravenous (0.8%)	Intravenous (2.1%)
	Nasal/inhalation (1.8%)	Transplacental (1.3%)	Nasal/inhalation (0%)	Intrauterine (0.6%)	Intravenous (4.6%)	Oral (3.6%)	Nasal/inhalation (2.5%)	Nasal/inhalation (1.0%)	Oral (2.5%)	Oral (7.3%)	Parenteral (0.3%)	Transplacental (1.0%)
	Intravenous (0.6%)	Intravenous (0.6%)	Intravenous (0%)		Parenteral (3.7%)	Intrathecal (1.4%)	Parenteral (0.4%)	Transplacental (0.5%)	Parenteral (2.5%)	Parenteral (7.3%)		Oropharyngeal (0.5%)
	Rectal (0.2%)	Nasal/inhalation (0.4%)	Rectal (0%)		Nasal/inhalation (3.1%)	Topical (1.1%)	Rectal (0%)	Parenteral (0.3%)		Subcutaneous (5.5%)		Intramuscular (0.3%)
Fatal outcome (%)	69.50%	29.70%	24.50%	32.70%	46.80%	21.00%	31.30%	36.90%	1.50%	13.40%	21.70%	22.40%
**Most important concomitant prescription psychotropic drugs recorded**												
Antidepressants (%)	20.90%	23.40%	9.40%	47.10%	14.30%	11.10%	13.70%	13.20%	1.50%	9.80%	17.60%	26.60%
Antipsychotics (%)	5.20%	6.60%	9.40%	21.40%	2.70%	2.90%	3.30%	4.10%	1.50%	7.10%	3.20%	6.60%
Benzodiazepines (%)	31.20%	19.60%	24.50%	35.10%	18.20%	13.60%	23.00%	18.80%	5.10%	27.70%	15.40%	18.20%
Gabapentinoids (%)	2.20%	9.40%	1.90%	20.30%	5.00%	5.60%	3.20%	6.20%	0.70%	1.80%	4.30%	12.30%
Mood Stabilizers (%)	2.00%	5.20%	0%	12.30%	2.20%	2.20%	1.60%	2.50%	0.70%	1.80%	2.40%	5.40%
OTCs (%):												
Anticholinergics (%)	1.40%	2.50%	3.40%	1.60%	0.70%	2.20%	0.40%	1.20%	0%	9.80%	0.90%	2.70%
Antihistamines (%)	19.70%	12.10%	9.40%	0%	6.00%	3.70%	8.70%	5.30%	5.10%	33.90%	5.60%	9.00%
Dextromethorphan (%)	12.50%	3.00%	0%	0.30%	0.70%	0.20%	1.50%	0.60%	0%	0%	1.50%	0.40%
Loperamide (%)	0%	0.80%	0%	0.30%	0.10%	0.10%	0.10%	0.20%	0%	0.90%	0.20%	0.50%
Paracetamol/Acetaminophen (%)	14.30%	17.50%	3.80%	25.10%	3.00%	2.70%	5.50%	5.70%	2.20%	8.90%	5.80%	14.00%
Pseudoephedrine and Pseudoephedrine-Containing Products (%)	0.40%	0.90%	0%	0%	0.10%	0%	0.30%	0.20%	0%	0%	0.10%	0.20%
Other Opioids (%)	67.60%	39.70%	20.80%	37.40%	21.50%	43.00%	31.00%	22.80%	5.90%	14.30%	16.60%	16.70%
Z-Drugs (%)	4.20%	4.10%	3.80%	2.40%	2.70%	2.10%	2.50%	2.90%	0.70%	5.40%	2.60%	5.60%
**Most important concomitant recreational drugs recorded**												
Alcohol (%)	8.10%	3.60%	11.30%	8.70%	3.10%	0.90%	8.70%	4.20%	2.20%	0.90%	3.60%	2.60%
Amphetamines and Methamphetamines (%)	4.50%	2.80%	3.40%	1.90%	1.70%	0.40%	3.80%	1.70%	0%	0%	1.50%	0.90%
Cannabis and Cannabinoids (%)	2.70%	1.00%	0%	0.50%	1.10%	0.30%	4.70%	1.80%	0.70%	0.90%	1.50%	0.50%
Cocaine (%)	19.30%	4.40%	1.90%	0.70%	3.50%	0.80%	8.80%	3.20%	0%	0%	2.60%	0.90%
Hallucinogens (%)	2.00%	0.60%	0%	0.70%	0.10%	0.10%	0.90%	0.40%	0%	0%	0.70%	0.20%
Heroin (%)	0%	9.10%	0%	4.00%	0%	1.00%	0%	1.80%	0%	0.90%	0%	0.40%
Ketamine (%)	0.40%	0.10%	0%	0.30%	0.20%	0.30%	0.20%	0.10%	0%	0%	0%	0.20%
NPS (%)	0%	0.10%	0%	0%	0%	0.00%	0%	0.00%	0%	0%	0.20%	0.10%

Abbreviations: ADR, adverse drug reaction; AE, adverse event; EMA, European Medicines Agency; FAERS, Food and Drug Administration Adverse Event Reporting System; NPS, new psychoactive substances; OTC, over-the-counter drugs; ROA, route of administration; SD, standard deviation; UK, United Kingdom; US, United States. Note: Full descriptive information on ADR reports can be found in Supplementary Table S1.

**Table 2:** Proportional reporting ratio signal scores regarding abuse/dependence and withdrawal issues for selected opioid drugs (European Medicines Agency EudraVigilance and the Food and Drug Administration (FDA) Adverse Event Reporting System datasets).

Preferred Term (PT)	Codeine	Dihydrocodeine	Fentanyl	Oxycodone	Pentazocine	Tramadol
	PRR (FDR)	PRR (FDR)	PRR (FDR)	PRR (FDR)	PRR (FDR)	PRR (FDR)
**Misuse-/Abuse-Related Terms**						
**Drug Abuse**						
EV	1.94 (<0.01)	0.90 (0.44)	0.93 (0.71)	0.91 (0.70)	2.23 (<0.01)	1.01 (0.02)
FAERS	1.96 (<0.01)	0.32 (0.41)	0.40 (0.43)	2.48 (<0.01)	1.17 (0.05)	0.62 (0.43)
**Drug Abuser**						
EV	NA	NA	0.31 (0.68)	2.52 (<0.01)	NA	0.65 (0.49)
FAERS	0.17 (0.42)	NA	0.13 (0.43)	10.17 (<0.01)	NA	0.29 (0.43)
**Drug Diversion**						
EV	0.88 (0.26)	NA	2.30 (<0.01)	0.72 (0.68)	NA	0.18 (0.71)
FAERS	NA	2.12 (<0.01) *	1.70 (<0.01)	1.17 (<0.01)	NA	0.25 (0.42)
**Drug Use Disorder**						
EV	NA	NA	NA	NA	NA	NA
FAERS	NA	NA	1.25 (0.07)	NA	NA	2.81 (<0.01) *
**Intentional Product Misuse**						
EV	2.23 (<0.01)	1.35 (<0.01) *	2.20 (<0.01)	0.33 (0.70)	0.34 (0.68)	1.24 (<0.01)
FAERS	1.25 (<0.01)	1.18 (0.03) *	1.09 (<0.01)	1.07 (<0.01)	NA	0.72 (0.42)
**Substance Abuse**						
EV	1.11 (<0.01) *	NA	0.09 (0.70)	8.84 (<0.01)	NA	0.14 (0.70)
FAERS	0.91 (0.23)	0.70 (0.25)	0.03 (0.43)	17.61 (<0.01)	NA	0.13 (0.43)
**Substance Use**						
EV	NA	NA	NA	NA	NA	NA
FAERS	NA	NA	0.53 (0.31)	NA	NA	3.51 (<0.01)
**Dependence-Related Terms**						
**Dependence**						
EV	0.92 (0.27)	NA	1.13 (<0.01)	0.17 (0.70)	NA	5.38 (<0.01)
FAERS	0.98 (0.14)	NA	0.92 (0.23)	0.64 (0.39)	NA	1.88 (<0.01)
**Drug Dependence**						
EV	0.78 (0.69)	1.24 (<0.01) *	0.21 (0.70)	2.75 (<0.01)	0.70 (0.52)	0.99 (0.22)
FAERS	0.24 (0.43)	0.30 (0.40)	0.09 (0.43)	11.53 (<0.01)	1.56 (<0.01) *	0.31 (0.43)
**Substance Dependence**						
EV	NA	NA	0.13 (0.70)	13.19 (<0.01)	NA	NA
FAERS	NA	NA	0.04 (0.42)	53.88 (<0.01)	NA	NA
**Withdrawal-Related Terms**						
**Drug Withdrawal Syndrome**						
EV	0.22 (0.70)	0.81 (0.39)	0.66 (0.70)	1.92 (<0.01)	0.57 (0.55)	0.65 (0.70)
FAERS	0.19 (0.42)	NA	0.68 (0.43)	2.82 (<0.01)	NA	0.32 (0.43)
**Overdose and Off-Label-Use Terms**						
**Intentional Overdose**						
EV	1.68 (<0.01) *	NA	0.47 (0.71)	0.53 (0.71)	NA	4.00 (<0.01)
FAERS	2.03 (<0.01)	2.39 (<0.01)	0.14 (0.43)	1.48 (<0.01)	NA	2.49 (<0.01)
**Off-Label Use**						
EV	0.88 (0.24)	NA	4.67 (<0.01)	0.28 (0.70)	NA	0.37 (0.71)
FAERS	0.59 (0.40)	1.70 (<0.01)	2.74 (<0.01)	0.44 (0.43)	NA	0.57 (0.42)
**Overdose**						
EV	0.93 (0.32)	1.78 (<0.01) *	1.02 (0.02)	0.77 (0.70)	NA	1.55 (<0.01)
FAERS	0.96 (0.23)	1.53 (<0.01)	0.51 (0.43)	2.24 (<0.01)	NA	0.72 (0.42)

* PRR is significant, but all four signal measures are not significant. Boldface denotes significant signals based on FDR < 0.05 for all four pharmacovigilance measures; minimum number of events to compute signal statistics is five for all measures. EV, EudraVigilance; FAERS, Food and Drug Administration Adverse Event Reporting System; FDR, false discovery rate; NA, not available (less than five events for this pair); PRR = proportional reporting ratio. Note: The full version of this table, including the reporting odds ratio, information component, and empirical Bayesian geometric mean, can be found in Supplementary Table S2.

## Data Availability

Restrictions apply to the availability of the EudraVigilance data. Data was obtained and are available by request from the European Medicines Agency. The FDA Adverse Event Reporting System data are publicly available and can be found here: https://www.fda.gov/drugs/questions-and-answers-fdas-adverse-event-reporting-system-faers/fda-adverse-event-reporting-system-faers-public-dashboard (accessed on 6 March 2022).

## Supplementary Materials

The following supporting information can be downloaded at https://www.mdpi.com/article/10.3390/ph15060675/s1. Table S1: Analysis of opioid-related adverse drug reaction reports recorded in the European Medicines Agency (EMA) EudraVigilance (EV) dataset and the Food and Drug Administration (FDA) Adverse Event Reporting System. Table S2: Signal scores regarding abuse/dependence and withdrawal issues for selected opioid drugs (European Medicines Agency EudraVigilance and the Food and Drug Administration (FDA) Adverse Event Reporting System (FAERS) datasets). Table S3: Signal scores regarding adverse drug reactions other than abuse/dependence and withdrawal issues for selected opioid drugs (European Medicines Agency/EMA and the Food and Drug Administration (FDA) Adverse Event Reporting System/FAERS datasets). Table S4: Pharmacovigilance signals of opioid-related adverse drug reaction reports compared to benzodiazepine-related reports in the Food and Drug Administration Adverse Event Reporting System (FAERS). Table S5: Description of cases involving opioids and new psychoactive substances (NPSs) recorded in both the European Medicines Agency (EMA) and the Food and Drug Administration Adverse Event Reporting System (FAERS) databases. Appendix SA: Materials and Methods. References [32,63,73,74,75,76,77,78,79,84,85,86,87] are cited in the supplementary materials.
