# Supplementary material for: Pharmacovigilance Signals of the Opioid Epidemic over 10 Years: Data Mining Methods in the Analysis of Pharmacovigilance Datasets Collecting Adverse Drug Reactions (ADRs) Reported to EudraVigilance (EV) and the FDA Adverse Event Reporting System (FAERS)
Source: Pharmaceuticals (Basel). 2022 May 27;15(6):675. doi: 10.3390/ph15060675 (PMC9231103; doi:10.3390/ph15060675)
Supplement: Supplementary file 1 [file pharmaceuticals-15-00675-s001.zip › Appendix SA.pdf]

Appendix SA

Materials and Methods

4.1 Data sources

The EMA is responsible for the EudraVigilance (EV) recording of ADRs reported for all medicinal products authorised in the European Economic Area (EEA) [73]. For the present study, access to data regarding cases related to the selected opioids submitted to EV up to April 2018 was formally requested from the EMA [73]. All reports included cases where codeine, dihydrocodeine, fentanyl, oxycodone, pentazocine, and tramadol were reported as a suspected or interacting active substance. Preferred terms (PT) for the present analysis were selected from the standardised Medical Dictionary for Regulatory Activities (MedDRA) Query (SMQ) 'Drug abuse, dependence and withdrawal' [84] including: 'Drug abuse', 'Substance abuse', 'Intentional product misuse', 'Dependence', 'Drug withdrawal syndrome', 'Withdrawal', 'Withdrawal syndrome'. PTs that may be indicative of an abuse event (described in detail in [85]) were also examined in this analysis.

Similarly, the FAERS, designed to support the FDA's post-marketing safety that contains information on adverse event and medication error reports submitted to the FDA [74], was queried in April 2018 for ADRs related to the selected opioids. FAERS data were available through the FAERS Public Dashboard and quarterly data extract files [74]. To enable a clearer comparison between EV and FAERS, we used the same timeframe for both datasets in the present analysis; therefore, any ADRs occurring in FAERS prior to February 2003 (the date of the first EV ADR for one of the six opioids under investigation herein) were removed from the analysis. Benzodiazepines ADR reports, pulled from FAERS in May 2022, were also restricted to this timeframe. In the present study, *abuse* was defined here as 'the intentional, non-therapeutic use by a patient or consumer of a product, OTC or prescription, for a perceived reward or desired non-therapeutic effect including, but not limited to, getting high (euphoria)'; *dependence* was intended the 'overwhelming desire by a patient or consumer to take a drug for non-therapeutic purposes together with inability to control or stop its use despite harmful consequences' [84]; and *withdrawal* was identified here in association with the abrupt cessation or reduction in intake of a drug in a habituated person, resulting in a substance-specific syndrome, with symptoms varying according to the psychoactive substance used and generally opposite the acute effects of drug [32].

4.2 Data Analysis

We performed a descriptive analysis of ADR report characteristics including sociodemographics, country of origin, most common diagnoses, route of administration, and concomitant licit/illicit substances. SPSS v28 was used for all descriptive analysis. Pharmacovigilance signal measures, including the reporting odds ratio (ROR), proportional reporting ratio (PRR), information component (IC), and empirical Bayesian geometric mean (EBGM) were calculated in each dataset using the R package PhViD [75]. All four pharmacovigilance measures were calculated due to differences in their sensitivity and early detection potential [76-77]; for brevity, only the PRR is shown in the text; all calculated measures can be found in the supplemental tables. Signals are disproportionality measures based on a 2 x 2 contingency table; they help determine whether a drug adverse event pair occurs more often than expected by comparing signal values to published thresholds [63,86]. Given the support for the use of the false discovery rate (FDR) to identify signals over thresholds, we used an FDR <0.05 to denote significance [78-79,87]. When significant signals are reported in this analysis, all four measures met significance criteria.
