# Supplementary material for: Pharmacovigilance Signals of the Opioid Epidemic over 10 Years: Data Mining Methods in the Analysis of Pharmacovigilance Datasets Collecting Adverse Drug Reactions (ADRs) Reported to EudraVigilance (EV) and the FDA Adverse Event Reporting System (FAERS)
Source: Pharmaceuticals (Basel). 2022 May 27;15(6):675. doi: 10.3390/ph15060675 (PMC9231103; doi:10.3390/ph15060675)
Supplement: Supplementary file 1 [file pharmaceuticals-15-00675-s001.zip › TableS1_all results general data_R1.pdf]

	CODEINE		DIHYDROCODEINE		FENTANYL		OXYCODONE		PENTAZOCINE		TRAMADOL	
	**EMA**	**FAERS**	**EMA**	**FAERS**	**EMA**	**FAERS**	**EMA**	**FAERS**	**EMA**	**FAERS**	**EMA**	**FAERS**
**Individual cases**	**814**	**6,764**	**53**	**575**	**5,443**	**54,640**	**7,441**	**45,672**	**136**	**112**	**2,619**	**22,530**
**Mean Age in years (SD)**	38.3 (13.6)	50.7 (19.6)	37.9 (12.7)	43.4 (22.2)	43.3 (16.0)	53.2 (19.2)	38.0 (13.6)	45.6 (18.2)	46.3 (16.5)	51.4 (21.1)	42.7 (15.7)	52.8 (20.4)
**M (%)**<br>**F (%)**	73.8% (540)<br>26.2% (192)	32.2% (1,983)<br>67.8% (4,167)	36.2% (17)<br>63.8% (30)	48.2% (244)<br>51.8% (262)	53.0% (2,459)<br>47.0% (2,178)	40.5% (19,354)<br>59.5% (28,382)	61.4% (3,929)<br>38.6% (2,468)	54.2% (22,504)<br>45.8% (19,036)	20.7% (28)<br>79.3% (107)	51.9% (54)<br>48.1% (50)	48.9% (1,142)<br>51.1% (1,195)	38.7% (7,890)<br>61.3% (12,479)
**Country of origin (five most recorded countries, %)**	US (58.6%)<br>Germany (12.1%)<br>France (7.4%)<br>Canada (6.5%)<br>Australia (4.7%)	US (67.1%)<br>UK (10.8%)<br>Canada (5.4%)<br>Australia (3.6%)<br>Norway (2.6%)	UK (31.8%)<br>Germany (22.7%)<br>France (18.2%)<br>Austria (9.1%)<br>New Zealand (6.8%)	UK (78.0%)<br>US (8.0%)<br>Japan (4.5%)<br>Italy (2.4%)<br>Germany (1.6%)<br>New Zealand (1.6%)	US (51.8%)<br>Canada (22.8%)<br>Germany (8.3%)<br>France (4.9%)<br>Estonia (2.2%)	US (71.3%)<br>Japan (8.6%)<br>France (3.9%)<br>UK (3.0%)<br>Australia (2.1%)	US (74.9%)<br>Canada (10.5%)<br>Australia (9.6%)<br>France (1.3%)<br>Germany (1.2%)	US (86.8%)<br>France (2.6%)<br>Japan (2.1%)<br>Canada (1.9%)<br>UK (1.3%)<br>Australia (1.3%)	Canada (63.0%)<br>US (18.5%)<br>India (10.9%)<br>Japan (4.2%)<br>UK (0.8%)	Japan (65.6%)<br>US (14.0%)<br>India (12.9%)<br>Turkey (2.2%)<br>Germany (1.1%)	US (48.4%)<br>Germany (13.3%)<br>France (7.6%)<br>Denmark (4.6%)<br>Sweden (3.8%)	US (48.9%)<br>France (16.8%)<br>UK (11.9%)<br>Germany (2.5%)<br>Italy (2.3%)
**Most common indications recorded for the index opioid when reported (%)**	-Drug abuse (1.9%)<br>-Pain (1.6%)<br>-Cough (1.4%)<br>-Headache (1.0%)<br>-Drug dependence (1.0%)<br>-Back pain (0.8%)<br>-Intentional product misuse (0.8%)<br>-Overdose (0.6%)<br>-Analgesic pain (0.6%)<br>-Migraine (0.6%)<br>-Withdrawal (0.2%)	-Pain (7.2%)<br>-Rheumatoid Arthritis (4.9%)<br>-Cough (2.6%)<br>-Analgesic therapy (1.8%)<br>-Back Pain (1.6%)<br>-Migraine (1.4%)<br>-Asthma (1.4%)<br>-Anxiety (1.4%)<br>-Drug Abuse (1.0%)<br>-Headache (1.0%)<br>-Drug Dependence (1.0%)	-Pain (20.0%)<br>-Procedural pain (10.0%)<br>-Drug dependence (6.7%)<br>-Toothache (3.3%)<br>-Headache (3.3%)<br>-Drug abuser (3.3%)<br>-Alcohol abuse (3.3%)<br>-Analgesic therapy (3.3%)<br>-Drug withdrawal maintenance therapy (3.3%)	-Pain (12.3%)<br>-Back Pain (5.9%)<br>-Rheumatoid arthritis (5.4%)<br>-Cough (2.5%)<br>-Psoriatic arthropathy (1.9%)<br>-Neuralgia (1.9%)<br>-Drug withdrawal syndrome (0.6%)	-Pain (25.0%)<br>-Intentional product misuse (7.3%)<br>-Back pain (4.7%)<br>-Drug abuse (2.2%)<br>-Cancer pain (2.0%)<br>-Breakthrough pain (1.2%)<br>-Sedation (1.0%)<br>-Arthralgia (1.0%)<br>-Fibromyalgia (0.9%)<br>-Analgesic therapy (0.9%)<br>-Neuralgia (0.6%)<br>-Drug withdrawal syndrome (0.3%)	-Pain (31.0%)<br>-Back pain (9.1%)<br>-Cancer pain (6.2%)<br>-Breakthrough pain (4.2%)<br>-Anaesthesia (2.0%)<br>-Fibromyalgia (1.9%)<br>-Arthralgia (1.7%)<br>-Induction of anaesthesia (1.6%)<br>-Analgesic Therapy (1.6%)<br>-Pain Management (1.4%)<br>-General Anaesthesia (1.3%)<br>-Drug abuse (0.4%)	-Drug abuse (15.3%)<br>-Pain (13.8%)<br>-Back pain (4.7%)<br>-Intentional product misuse (3.5%)<br>-Drug abuser (1.2%)<br>-Drug dependence (0.6%)<br>-Arthralgia (0.5%)<br>-Neck pain (0.5%)<br>-Procedural pain (0.4%)	-Pain (30.5%)<br>-Back Pain (5.8%)<br>-Drug abuse (4.0%)<br>-Cancer Pain (3.5%)<br>-Breakthrough pain (2.2%)<br>-Drug abuser (1.3%)<br>-Analgesic therapy (1.2%)<br>-Arthralgia (1.0%)<br>-Fibromyalgia (1.0%)<br>-Neuralgia (0.9%)<br>-Procedural pain (0.7%)<br>-Rheumatoid arthritis (0.7%)<br>-Drug dependence (0.6%)	-Pain (24.4%)<br>-Drug abuse (7.7%)<br>-Migraine (3.8%)<br>-Abdominal pain (2.6%)<br>-Analgesic therapy (2.6%)<br>-Substance abuse (1.3%)<br>-Dependence (1.3%)	-Pain (17.3%)<br>-Analgesic therapy (14.3%)<br>-Drug abuse (8.2%)<br>-Induction of anaesthesia (5.1%)<br>-Cancer pain (5.1%)<br>-Sedation (4.1%)<br>-Maintenance of anaesthesia (3.1%)<br>-Back Pain (3.1%)<br>-Migraine (3.1%)<br>-Procedural pain (2.0%)<br>-Anaesthetic premedication (2.0%)	-Pain (18.9%)<br>-Back pain (7.8%)<br>-Headache (2.7%)<br>-Arthralgia (2.2%)<br>-Drug abuse (1.7%)<br>-Procedural pain (1.0%)<br>-Migraine (1.0%)<br>-Fibromyalgia (1.0%)	-Pain (21.6%)<br>-Back pain (6.8%)<br>-Depression (6.1%)<br>-Fibromyalgia (2.1%)<br>-Analgesic therapy (2.0%)<br>-Rheumatoid arthritis (1.9%)<br>-Neuralgia (1.7%)<br>-Osteoarthritis (1.4%)<br>-Headache (1.4%)<br>-Arthritis (1.4%)<br>-Procedural pain (1.0%)<br>-Migraine (0.8%)<br>-Neck pain (0.7%)<br>-Neuropathy peripheral (0.7%)

										-Headache (2.0%)		-Musculoskeletal pain (0.6%)<br>-Muscle spasms (0.6%)<br>-Postoperative analgesia (0.4%)<br>-Psoriatic arthropathy (0.3%)<br>-Pain management (0.3%)<br>-Muscle relaxant therapy (0.3%)<br>-Diabetic neuropathy (0.2%)<br>-Post herpetic neuralgia (0.2%)<br>-Drug abuse (0.2%)
**ROA (%)**	Oral (26.9%)<br>Parenteral (9.0%)<br>Nasal/inhalation (1.8%)<br>Intravenous (0.6%)<br>Rectal (0.2%)	Oral (32.2%)<br>Parenteral (2.3%)<br>Transplacental (1.3%)<br>Intravenous (0.6%)<br>Nasal/inhalation (0.4%)	Oral (63.0%)<br>Parenteral (0%)<br>Nasal/inhalation (0%)<br>Intravenous (0%)<br>Rectal (0%)	Oral (40.1%)<br>Transplacental (16.5%)<br>Intrauterine (0.6%)	Transdermal (44.9%)<br>Oral (22.6%)<br>Intravenous (4.6%)<br>Parenteral (3.7%)<br>Nasal/inhalation (3.1%)	Transdermal (75.0%)<br>Intravenous (6.0%)<br>Oral (3.6%)<br>Intrathecal (1.4%)<br>Topical (1.1%)	Oral (56.0%)<br>Intravenous (3.2%)<br>Nasal/inhalation (2.5%)<br>Parenteral (0.4%)<br>Rectal (0%)	Oral (76.1%)<br>Intravenous (1.3%)<br>Nasal/inhalation (1.0%)<br>Transplacental (0.5%)<br>Parenteral (0.3%)	Intravenous (70.0%)<br>Intramuscular (19.2%)<br>Oral (2.5%)<br>Parenteral (2.5%)	Intramuscular (32.7%)<br>Intravenous (32.7%)<br>Oral (7.3%)<br>Parenteral (7.3%)<br>Subcutaneous (5.5%)	Oral (86.5%)<br>Intravenous (0.8%)<br>Parenteral (0.3%)	Oral (63.9%)<br>Intravenous (2.1%)<br>Transplacental (1.0%)<br>Oropharyngeal (0.5%)<br>Intramuscular (0.3%)
**Fatal outcome (%)**	69.5% (566)	29.7% (2010)	24.5% (13)	32.7% (188)	46.8% (2546)	21.0% (11,469)	31.3% (2330)	36.9% (16,841)	1.5% (2)	13.4% (15)	21.7% (568)	22.4% (5054)
**Most important concomitant prescription psychotropic drugs recorded**												
**ANTIDEPRESSANTS (%)**	20.9% (170)	23.4% (1582)	9.4% (5)	47.1% (271)	14.3% (781)	11.1% (6051)	13.7% (1022)	13.2% (6032)	1.5% (2)	9.8% (11)	17.6% (461)	26.6% (5982)
**ANTIPSYCHOTICS (%)**	5.2% (42)	6.6% (445)	9.4% (5)	21.4% (123)	2.7% (149)	2.9% (1697)	3.3% (245)	4.1% (1850)	1.5% (2)	7.1% (8)	3.2% (85)	6.6% (1485)
**BENZODIAZEPINES (%)**	31.2% (254)	19.6% (1323)	24.5% (13)	35.1% (202)	18.2% (992)	13.6% (7423)	23.0% (1711)	18.8% (8587)	5.1% (7)	27.7% (31)	15.4% (403)	18.2% (4110)
**GABAPENTINOIDS (%)**	2.2% (18)	9.4% (637)	1.9% (1)	20.3% (117)	5.0% (273)	5.6% (3086)	3.2% (235)	6.2% (2817)	0.7% (1)	1.8% (2)	4.3% (112)	12.3% (2781)

**MOOD STABILIZERS (%)**	2.0% (16)	5.2% (354)	0% (0)	12.3% (71)	2.2% (121)	2.2% (1188)	1.6% (118)	2.5% (1133)	0.7% (1)	1.8% (2)	2.4% (64)	5.4% (1213)
**OTCs (%):**												
Anticholinergics (%)	1.4% (11)	2.5% (167)	3.4% (2)	1.6% (9)	0.7% (37)	2.2% (1190)	0.4% (33)	1.2% (533)	0% (0)	9.8% (11)	0.9% (23)	2.7% (609)
Antihistamines (%)	19.7% (160)	12.1% (820)	9.4% (5)	0% (0)	6.0% (325)	3.7% (2042)	8.7% (495)	5.3% (2398)	5.1% (7)	33.9% (38)	5.6% (147)	9.0% (2032)
Dextrometorphan (%)	12.5% (102)	3.0% (200)	0% (0)	0.3% (2)	0.7% (37)	0.2% (96)	1.5% (112)	0.6% (268)	0% (0)	0% (0)	1.5% (26)	0.4% (95)
Loperamide (%)	0% (0)	0.8% (51)	0% (0)	0.3% (2)	0.1% (4)	0.1% (63)	0.1% (11)	0.2% (92)	0% (0)	0.9% (1)	0.2% (6)	0.5% (106)
Paracetamol/Acetaminophen (%)	14.3% (116)	17.5% (1186)	3.8% (2)	25.1% (147)	3.0% (165)	2.7% (1491)	5.5% (411)	5.7% (2612)	2.2% (3)	8.9% (10)	5.8% (151)	14.0% (3143)
Pseudoephedrine and pseudoephedrine-containing products (%)	0.4% (3)	0.9% (63)	0% (0)	0% (0)	0.1% (3)	0.0% (26)	0.3% (26)	0.2% (72)	0% (0)	0% (0)	0.1% (3)	0.2% (56)
**OTHER OPIOIDS (%)**	67.6% (550)	39.7% (2688)	20.8% (11)	37.4% (215)	21.5% (1172)	43.0% (23,490)	31.0% (2304)	22.8% (10,392)	5.9% (8)	14.3% (16)	16.6% (436)	16.7% (3755)
**Z-DRUGS (%)**	4.2% (34)	4.1% (279)	3.8% (2)	2.4% (14)	2.7% (145)	2.1% (1201)	2.5% (184)	2.9% (1341)	0.7% (1)	5.4% (6)	2.6% (68)	5.6% (1270)
**Most important concomitant recreational drugs recorded**												
**ALCOHOL (%)**	8.1% (66)	3.6% (246)	11.3% (6)	8.7% (50)	3.1% (168)	0.9% (475)	8.7% (645)	4.2% (1929)	2.2% (3)	0.9% (1)	3.6% (94)	2.6% (595)
**AMPHETAMINES AND METAMPHETAMINES (%)**	4.5% (37)	2.8% (192)	3.4% (2)	1.9% (11)	1.7% (95)	0.4% (241)	3.8% (284)	1.7% (783)	0% (0)	0% (0)	1.5% (38)	0.9% (208)
**CANNABIS and CANNABINOIDS (%)**	2.7% (22)	1.0% (64)	0% (0)	0.5% (3)	1.1% (58)	0.3% (164)	4.7% (346)	1.8% (803)	0.7% (1)	0.9% (1)	1.5% (40)	0.5% (97)
**COCAINE (%)**	19.3% (149)	4.4% (296)	1.9% (1)	0.7% (4)	3.5% (190)	0.8% (421)	8.8% (652)	3.2% (1481)	0% (0)	0% (0)	2.6% (69)	0.9% (196)
**HALLUCINOGENS (%)**	2.0% (16)	0.6% (43)	0% (0)	0.7% (4)	0.1% (5)	0.1% (31)	0.9% (70)	0.4% (166)	0% (0)	0% (0)	0.7% (18)	0.2% (41)
**HEROIN (%)**	0% (0)	9.1% (614)	0% (0)	4.0% (23)	0% (0)	1.0% (542)	0% (0)	1.8% (804)	0% (0)	0.9% (1)	0% (0)	0.4% (94)
**KETAMINE (%)**	0.4% (3)	0.1% (7)	0% (0)	0.3% (2)	0.2% (9)	0.3% (177)	0.2% (13)	0.1% (30)	0% (0)	0% (0)	0% (0)	0.2% (48)
**NPS (%)**	0% (0)	0.1% (5)	0% (0)	0% (0)	0% (0)	0.0% (5)	0% (0)	0.0% (8)	0% (0)	0% (0)	0.2% (4)	0.1% (19)

Table S1. Analysis of opioid-related adverse drug reaction reports recorded in the European Medicines Agency (EMA) EudraVigilance (EV) dataset and the Food and Drug Administration (FDA) Adverse Event Reporting System

Abbreviations: AE: Adverse Event; EMA: European Medicines Agency; FAERS: Food and Drug Administration Adverse Event Reporting System; NPS: new psychoactive substances; OTC: over the counter drugs; ROA: route of administration; SD: Standard Deviation; UK: United Kingdom; US: United States
