# Supplementary material for: Pharmacovigilance Signals of the Opioid Epidemic over 10 Years: Data Mining Methods in the Analysis of Pharmacovigilance Datasets Collecting Adverse Drug Reactions (ADRs) Reported to EudraVigilance (EV) and the FDA Adverse Event Reporting System (FAERS)
Source: Pharmaceuticals (Basel). 2022 May 27;15(6):675. doi: 10.3390/ph15060675 (PMC9231103; doi:10.3390/ph15060675)
Supplement: Supplementary file 1 [file pharmaceuticals-15-00675-s001.zip › TableS2_signals_R1.pdf]

Preferred terms (PT)	Codeine				Dihydrocodeine				Fentanyl				Oxycodone				Pentazocine				Tramadol			
	PRR	ROR	IC025	EB05	PRR	ROR	IC025	EB05	PRR	ROR	IC025	EB05	PRR	ROR	IC025	EB05	PRR	ROR	IC025	EB05	PRR	ROR	IC025	EB05
**Misuse/abuse-related terms**																								
**Drug abuse**																								
EV	**1.94 (<0.01)**	**2.29 (<0.01)**	**0.77 (<0.01)**	**1.73 (<0.01)**	0.90 (0.44)	0.88 (0.44)	-0.69 (0.17)	0.68 (0.20)	0.93 (0.71)	0.92 (0.71)	**-0.14 (0.03)**	**0.92 (0.04)**	0.91 (0.70)	0.90 (0.70)	**-0.12 (0.02)**	**0.93 (0.04)**	**2.23 (<0.01)**	**2.82 (<0.01)**	**0.82 (<0.01)**	**1.80 (<0.01)**	**1.01 (0.02)**	**1.01 (0.03)**	**-0.08 (0.01)**	**0.96 (0.02)**
FAERS	**1.96 (<0.01)**	**1.99 (<0.01)**	**0.79 (<0.01)**	**1.75 (<0.01)**	0.32 (0.41)	0.32 (0.41)	-2.40 (0.41)	0.23 (0.45)	0.40 (0.43)	0.39 (0.43)	-0.99 (0.25)	0.51 (0.29)	**2.48 (<0.01)**	**2.52 (<0.01)**	**0.66 (<0.01)**	**1.60 (<0.01)**	1.17 (0.05)	1.17 (0.05)	-0.75 (0.18)	0.67 (0.18)	0.62 (0.43)	0.62 (0.43)	-0.67 (0.17)	0.64 (0.20)
**Drug abuser**																								
EV	NA	NA	NA	NA	NA	NA	NA	NA	0.31 (0.68)	0.31 (0.68)	-2.50 (0.42)	0.32 (0.48)	**2.52 (<0.01)**	**2.52 (<0.01)**	**-0.15 (0.03)**	**1.02 (0.01)**	NA	NA	NA	NA	0.65 (0.49)	0.65 (0.49)	-1.87 (0.37)	0.45 (0.43)
FAERS	0.17 (0.42)	0.17 (0.42)	-3.03 (0.45)	0.14 (0.47)	NA	NA	NA	NA	0.13 (0.43)	0.13 (0.43)	-2.47 (0.41)	0.19 (0.46)	**10.17 (<0.01)**	**10.36 (<0.01)**	**1.22 (<0.01)**	**2.36 (<0.01)**	NA	NA	NA	NA	0.29 (0.43)	0.29 (0.43)	-1.77 (0.36)	0.30 (0.43)
**Drug diversion**																								
EV	0.88 (0.26)	0.88 (0.26)	-1.39 (0.30)	0.53 (0.34)	NA	NA	NA	NA	**2.30 (<0.01)**	**2.31 (<0.01)**	**0.35 (<0.01)**	**1.34 (<0.01)**	0.72 (0.68)	0.72 (0.68)	-0.63 (0.16)	0.70 (0.18)	NA	NA	NA	NA	0.18 (0.71)	0.18 (0.71)	-3.36 (0.47)	0.19 (0.49)
FAERS	NA	NA	NA	NA	**2.12 (<0.01)**	**2.12 (<0.01)**	-0.51 (0.12)	0.75 (0.13)	**1.70 (<0.01)**	**1.70 (<0.01)**	**0.17 (<0.01)**	**1.18 (<0.01)**	**1.17 (<0.01)**	**1.17 (<0.01)**	**-0.11 (0.01)**	**0.97 (0.01)**	NA	NA	NA	NA	0.25 (0.42)	0.25 (0.42)	-2.30 (0.40)	0.23 (0.45)
**Drug use disorder**																								
EV	NA	NA	NA	NA	NA	NA	NA	NA	NA	NA	NA	NA	NA	NA	NA	NA	NA	NA	NA	NA	NA	NA	NA	NA
FAERS	NA	NA	NA	NA	NA	NA	NA	NA	1.25 (0.07)	1.25 (0.07)	-1.23 (0.29)	0.60 (0.23)	NA	NA	NA	NA	NA	NA	NA	NA	**2.81 (<0.01)**	**2.81 (<0.01)**	-0.60 (0.15)	0.80 (0.09)
**Intentional product misuse**																								
EV	**2.23 (<0.01)**	**2.35 (<0.01)**	**0.86 (<0.01)**	**1.82 (<0.01)**	**1.35 (<0.01)**	**1.37 (<0.01)**	-0.42 (0.10)	0.80 (0.11)	**2.20 (<0.01)**	**2.28 (<0.01)**	**0.58 (<0.01)**	**1.53 (<0.01)**	0.33 (0.70)	0.31 (0.70)	-1.15 (0.26)	0.46 (0.42)	0.34 (0.68)	0.33 (0.68)	-2.73 (0.43)	0.28 (0.48)	**1.24 (<0.01)**	**1.26 (<0.01)**	**0.11 (<0.01)**	**1.10 (<0.01)**
FAERS	**1.25 (<0.01)**	**1.25 (<0.01)**	**0.05 (<0.01)**	**1.06 (0.01)**	**1.18 (0.03)**	**1.18 (0.03)**	-0.54 (0.13)	0.75 (0.13)	**1.09 (<0.01)**	**1.09 (<0.01)**	**-0.03 (0.01)**	**1.00 (0.01)**	**1.07 (<0.01)**	**1.07 (<0.01)**	**-0.05 (0.01)**	**0.99 (0.01)**	NA	NA	NA	NA	0.72 (0.42)	0.72 (0.42)	-0.55 (0.14)	0.70 (0.16)
**Substance abuse**																								
EV	**1.11 (<0.01)**	**1.12 (<0.01)**	**-0.23 (0.05)**	0.89 (0.05)	NA	NA	NA	NA	0.09 (0.70)	0.09 (0.70)	-3.31 (0.46)	0.11 (0.49)	**8.84 (<0.01)**	**9.26 (<0.01)**	**0.75 (<0.01)**	**1.72 (<0.01)**	NA	NA	NA	NA	0.14 (0.70)	0.13 (0.70)	-3.13 (0.45)	0.13 (0.49)
FAERS	0.91 (0.23)	0.91 (0.23)	-0.49 (0.12)	0.75 (0.13)	0.70 (0.25)	0.70 (0.25)	-1.67 (0.34)	0.42 (0.36)	0.03 (0.43)	0.03 (0.43)	-4.66 (0.49)	0.04 (0.47)	**17.61 (<0.01)**	**17.79 (<0.01)**	**1.28 (<0.01)**	**2.50 (<0.01)**	NA	NA	NA	NA	0.13 (0.43)	0.13 (0.43)	-3.02 (0.45)	0.13 (0.47)
**Substance use**																								
EV	NA	NA	NA	NA	NA	NA	NA	NA	NA	NA	NA	NA	NA	NA	NA	NA	NA	NA	NA	NA	NA	NA	NA	NA
FAERS	NA	NA	NA	NA	NA	NA	NA	NA	0.53 (0.31)	0.53 (0.31)	-1.91 (0.37)	0.40 (0.37)	NA	NA	NA	NA	NA	NA	NA	NA	**3.51 (<0.01)**	**3.51 (<0.01)**	**-0.07 (0.01)**	**1.06 (0.01)**
**Dependence-related terms**																								
**Dependence**																								
EV	0.92 (0.27)	0.92 (0.27)	-0.89 (0.21)	0.62 (0.25)	NA	NA	NA	NA	**1.13 (<0.01)**	**1.13 (<0.01)**	**-0.15 (0.03)**	**0.95 (0.02)**	0.17 (0.70)	0.16 (0.70)	-2.19 (0.39)	0.24 (0.49)	NA	NA	NA	NA	**5.38 (<0.01)**	**5.49 (<0.01)**	**1.46 (<0.01)**	**2.77 (<0.01)**
FAERS	0.98 (0.14)	0.98 (0.14)	-0.94 (0.23)	0.61 (0.23)	NA	NA	NA	NA	0.92 (0.23)	0.92 (0.23)	-0.42 (0.09)	0.80 (0.09)	0.64 (0.39)	0.64 (0.39)	-0.86 (0.22)	0.60 (0.23)	NA	NA	NA	NA	**1.88 (<0.01)**	**1.88 (<0.01)**	**0.29 (<0.01)**	**1.29 (<0.01)**
**Drug dependence**																								

EV	0.78 (0.69)	0.77 (0.69)	-0.69 (0.17)	0.66 (0.22)	**1.24 (<0.01)**	**1.25 (<0.01)**	-0.50 (0.12)	0.77 (0.14)	0.21 (0.70)	0.20 (0.70)	-2.05 (0.38)	0.25 (0.49)	**2.75 (<0.01)**	**2.89 (<0.01)**	**0.47 (<0.01)**	**1.42 (<0.01)**	0.70 (0.52)	0.69 (0.53)	-1.36 (0.30)	0.49 (0.39)	0.99 (0.22)	0.99 (0.22)	**-0.17 (0.04)**	**0.91 (0.05)**
FAERS	0.24 (0.43)	0.24 (0.43)	-2.34 (0.40)	0.21 (0.46)	0.30 (0.40)	0.29 (0.40)	-2.64 (0.43)	0.21 (0.46)	0.09 (0.43)	0.09 (0.43)	-2.99 (0.44)	0.13 (0.47)	**11.53 (<0.01)**	**11.91 (<0.01)**	**1.26 (<0.01)**	**2.43 (<0.01)**	**1.56 (<0.01)**	**1.57 (<0.01)**	-0.38 (0.08)	0.82 (0.08)	0.31 (0.43)	0.31 (0.43)	-1.61 (0.34)	0.33 (0.41)
**Substance dependence**																								
EV	NA	NA	NA	NA	NA	NA	NA	NA	0.13 (0.70)	0.13 (0.70)	-3.43 (0.47)	0.15 (0.49)	**13.19 (<0.01)**	**13.25 (<0.01)**	**0.57 (<0.01)**	**1.58 (<0.01)**	NA	NA	NA	NA	NA	NA	NA	NA
FAERS	NA	NA	NA	NA	NA	NA	NA	NA	0.04 (0.42)	0.04 (0.42)	-5.19 (0.50)	0.03 (0.47)	**53.88 (<0.01)**	**53.95 (<0.01)**	**1.18 (<0.01)**	**2.41 (<0.01)**	NA	NA	NA	NA	NA	NA	NA	NA
**Withdrawal-related terms**																								
**Drug withdrawal syndrome**																								
EV	0.22 (0.70)	0.22 (0.70)	-2.80 (0.43)	0.19 (0.49)	0.81 (0.39)	0.80 (0.39)	-1.42 (0.31)	0.51 (0.36)	0.66 (0.70)	0.65 (0.70)	-0.58 (0.14)	0.69 (0.19)	**1.92 (<0.01)**	**1.96 (<0.01)**	**0.30 (<0.01)**	**1.26 (<0.01)**	0.57 (0.55)	0.56 (0.56)	-1.87 (0.37)	0.41 (0.45)	0.65 (0.70)	0.64 (0.70)	-0.76 (0.19)	0.61 (0.26)
FAERS	0.19 (0.42)	0.19 (0.42)	-2.76 (0.43)	0.16 (0.46)	NA	NA	NA	NA	0.68 (0.43)	0.67 (0.43)	-0.45 (0.10)	0.75 (0.13)	**2.82 (<0.01)**	**2.85 (<0.01)**	**0.72 (<0.01)**	**1.67 (<0.01)**	NA	NA	NA	NA	0.32 (0.43)	0.32 (0.43)	-1.61 (0.34)	0.34 (0.41)
**Overdose and off label use terms**																								
**Intentional overdose**																								
EV	**1.68 (<0.01)**	**1.69 (<0.01)**	-0.33 (0.08)	0.82 (0.10)	NA	NA	NA	NA	0.47 (0.71)	0.47 (0.71)	-1.38 (0.30)	0.43 (0.43)	0.53 (0.71)	0.52 (0.71)	-0.97 (0.23)	0.57 (0.30)	NA	NA	NA	NA	**4.00 (<0.01)**	**4.02 (<0.01)**	**1.03 (<0.01)**	**2.02 (<0.01)**
FAERS	**2.03 (<0.01)**	**2.04 (<0.01)**	**0.69 (<0.01)**	**1.65 (<0.01)**	**2.39 (<0.01)**	**2.40 (<0.01)**	**0.47 (<0.01)**	**1.34 (<0.01)**	0.14 (0.43)	0.14 (0.43)	-2.41 (0.41)	0.20 (0.46)	**1.48 (<0.01)**	**1.48 (<0.01)**	**0.21 (<0.01)**	**1.19 (<0.01)**	NA	NA	NA	NA	**2.49 (<0.01)**	**2.50 (<0.01)**	**0.81 (<0.01)**	**1.80 (<0.01)**
**Off-label use**																								
EV	0.88 (0.24)	0.88 (0.24)	-1.39 (0.30)	0.53 (0.34)	NA	NA	NA	NA	**4.67 (<0.01)**	**4.70 (<0.01)**	**0.79 (<0.01)**	**1.80 (<0.01)**	0.28 (0.70)	0.28 (0.70)	-1.67 (0.34)	0.36 (0.47)	NA	NA	NA	NA	0.37 (0.71)	0.37 (0.71)	-2.11 (0.39)	0.32 (0.48)
FAERS	0.59 (0.40)	0.59 (0.40)	-1.19 (0.28)	0.47 (0.32)	**1.70 (<0.01)**	**1.71 (<0.01)**	**-0.14 (0.02)**	**0.94 (0.02)**	**2.74 (<0.01)**	**2.75 (<0.01)**	**0.56 (<0.01)**	**1.51 (<0.01)**	0.44 (0.43)	0.44 (0.43)	-1.06 (0.26)	0.49 (0.30)	NA	NA	NA	NA	0.57 (0.42)	0.57 (0.42)	-0.91 (0.23)	0.55 (0.26)
**Overdose**																								
EV	0.93 (0.32)	0.93 (0.32)	-0.58 (0.14)	0.72 (0.17)	**1.78 (<0.01)**	**1.81 (<0.01)**	-0.29 (0.06)	0.83 (0.10)	**1.02 (0.02)**	**1.02 (0.02)**	**-0.15 (0.03)**	**0.93 (0.04)**	0.77 (0.70)	0.77 (0.70)	-0.35 (0.08)	0.81 (0.11)	NA	NA	NA	NA	**1.55 (<0.01)**	**1.56 (<0.01)**	**0.31 (<0.01)**	**1.28 (<0.01)**
FAERS	0.96 (0.23)	0.96 (0.23)	**-0.21 (0.04)**	**0.88 (0.05)**	**1.53 (<0.01)**	**1.55 (<0.01)**	**0.23 (<0.01)**	**1.20 (<0.01)**	0.51 (0.43)	0.50 (0.43)	-0.72 (0.18)	0.61 (0.22)	**2.24 (<0.01)**	**2.28 (<0.01)**	**0.60 (<0.01)**	**1.53 (<0.01)**	NA	NA	NA	NA	0.72 (0.42)	0.71 (0.42)	-0.48 (0.11)	0.72 (0.14)

Table S2. Signal scores regarding abuse/dependence and withdrawal issues for selected opioid drugs (European Medicines Agency EudraVigilance and the Food and Drug Administration [FDA] Adverse Event Reporting System [FAERS] datasets)

Boldface denotes signals based on FDR<0.05; Minimum number of events to compute signal statistics = 5 for all measures.

EV: EudraVigilance; EB05: 5% quantile of the posterior distribution of the empirical Bayesian geometric mean (estimated FDR); FAERS: Food and Drug Administration Adverse Event Reporting System; FDR: false discovery rate; IC025: 2.5% quantile of the posterior distribution of information component (estimated FDR); NA: not available (less than 5 events for this pair); PRR: proportional reporting ratio (estimated FDR); ROR: reporting odds ratio (estimated FDR).
