# Supplementary material for: Pharmacovigilance Signals of the Opioid Epidemic over 10 Years: Data Mining Methods in the Analysis of Pharmacovigilance Datasets Collecting Adverse Drug Reactions (ADRs) Reported to EudraVigilance (EV) and the FDA Adverse Event Reporting System (FAERS)
Source: Pharmaceuticals (Basel). 2022 May 27;15(6):675. doi: 10.3390/ph15060675 (PMC9231103; doi:10.3390/ph15060675)
Supplement: Supplementary file 1 [file pharmaceuticals-15-00675-s001.zip › TableS3_signals_R1.pdf]

**Preferred terms (PT)**	**Codeine**				**Dihydrocodeine**				**Fentanyl**				**Oxycodone**				**Pentazocine**				**Tramadol**			
	PRR	ROR	IC025	EB05	PRR	ROR	IC025	EB05	PRR	ROR	IC025	EB05	PRR	ROR	IC025	EB05	PRR	ROR	IC025	EB05	PRR	ROR	IC025	EB05
**Acute Psychosis**																								
EMA	NA	NA	NA	NA	NA	NA	NA	NA	NA	NA	NA	NA	NA	NA	NA	NA	NA	NA	NA	NA	NA	NA	NA	NA
FAERS	NA	NA	NA	NA	NA	NA	NA	NA	1.03 (0.13)	1.04 (0.13)	-1.25 (0.29)	0.58 (0.24)	NA	NA	NA	NA	NA	NA	NA	NA	**3.07 (<0.01)**	**3.07 (<0.01)**	-0.31 (0.06)	**0.93 (0.03)**
**Aggression**																								
EMA	0.84 (0.31)	0.84 (0.31)	-1.56 (0.33)	0.50 (0.37)	NA	NA	NA	NA	0.48 (0.71)	0.48 (0.71)	-1.35 (0.30)	0.46 (0.42)	**1.96 (<0.01)**	**1.97 (<0.01)**	**0.06 (<0.01)**	**1.12 (<0.01)**	NA	NA	NA	NA	0.88 (0.38)	0.88 (0.38)	-0.81 (0.20)	0.65 (0.23)
FAERS	**1.14 (<0.01)**	**1.14 (0.02)**	-0.32 (0.06)	0.85 (0.06)	**1.33 (0.04)**	**1.33 (0.04)**	-1.00 (0.25)	0.60 (0.23)	0.33 (0.43)	0.33 (0.43)	-1.38 (0.31)	0.41 (0.37)	**1.90 (<0.01)**	**1.90 (<0.01)**	**0.35 (<0.01)**	**1.32 (<0.01)**	NA	NA	NA	NA	**1.42 (<0.01)**	**1.42 (<0.01)**	**0.16 (<0.01)**	**1.16 (<0.01)**
**Confusional state**																								
EMA	NA	NA	NA	NA	NA	NA	NA	NA	0.99 (0.23)	0.99 (0.23)	-0.44 (0.11)	0.80 (0.12)	0.87 (0.47)	0.87 (0.47)	-0.47 (0.12)	0.78 (0.13)	NA	NA	NA	NA	**1.40 (<0.01)**	**1.40 (<0.01)**	**-0.12 (0.02)**	**0.97 (0.02)**
FAERS	0.45 (0.42)	0.45 (0.42)	-1.47 (0.32)	0.38 (0.38)	**1.14 (0.05)**	**1.14 (0.05)**	-0.55 (0.13)	0.74 (0.13)	0.72 (0.42)	0.72 (0.42)	-0.40 (0.09)	0.77 (0.11)	0.83 (0.41)	0.83 (0.41)	-0.29 (0.05)	0.84 (0.07)	NA	NA	NA	NA	**2.10 (<0.01)**	**2.11 (<0.01)**	**0.69 (<0.01)**	**1.64 (<0.01)**
**Delirium**																								
EMA	NA	NA	NA	NA	NA	NA	NA	NA	0.96 (0.23)	0.96 (0.23)	-1.03 (0.24)	0.62 (0.25)	0.54 (0.62)	0.54 (0.62)	-1.41 (0.31)	0.50 (0.37)	NA	NA	NA	NA	**1.44 (<0.01)**	**1.44 (<0.01)**	-0.78 (0.19)	0.70 (0.18)
FAERS	0.43 (0.41)	0.43 (0.41)	-1.80 (0.36)	0.33 (0.41)	NA	NA	NA	NA	0.97 (0.22)	0.97 (0.22)	**-0.19 (0.03)**	**0.90 (0.03)**	**1.21 (<0.01)**	**1.21 (<0.01)**	**0.01 (<0.01)**	**1.04 (0.01)**	NA	NA	NA	NA	0.95 (0.23)	0.95 (0.23)	-0.29 (0.06)	0.85 (0.06)
**Euphoric mood**																								
EMA	0.47 (0.57)	0.47 (0.57)	-2.28 (0.40)	0.36 (0.47)	NA	NA	NA	NA	0.11 (0.70)	0.11 (0.70)	-3.48 (0.47)	0.13 (0.49)	**2.66 (<0.01)**	**2.68 (<0.01)**	**0.29 (<0.01)**	**1.29 (<0.01)**	NA	NA	NA	NA	**1.49 (<0.01)**	**1.49 (<0.01)**	**0.06 (<0.01)**	**1.09 (<0.01)**
FAERS	0.34 (0.40)	0.34 (0.40)	-2.34 (0.40)	0.25 (0.44)	NA	NA	NA	NA	0.33 (0.43)	0.33 (0.43)	-1.39 (0.31)	0.40 (0.37)	**4.41 (<0.01)**	**4.42 (<0.01)**	**0.85 (<0.01)**	**1.86 (<0.01)**	NA	NA	NA	NA	0.44 (0.42)	0.44 (0.42)	-1.37 (0.31)	0.41 (0.36)
**Feeling of relaxation**																								
EMA	NA	NA	NA	NA	NA	NA	NA	NA	NA	NA	NA	NA	NA	NA	NA	NA	NA	NA	NA	NA	NA	NA	NA	NA
FAERS	NA	NA	NA	NA	NA	NA	NA	NA	NA	NA	NA	NA	NA	NA	NA	NA	NA	NA	NA	NA	NA	NA	NA	NA
**Hallucinations, visual**																								
EMA	NA	NA	NA	NA	NA	NA	NA	NA	0.78 (0.40)	0.78 (0.40)	-1.43 (0.31)	0.53 (0.34)	0.41 (0.69)	0.41 (0.69)	-1.86 (0.36)	0.42 (0.45)	NA	NA	NA	NA	**3.63 (<0.01)**	**3.64 (<0.01)**	**0.20 (<0.01)**	**1.10 (<0.01)**
FAERS	**1.26 (<0.01)**	**1.26 (<0.01)**	-0.34 (0.07)	0.84 (0.06)	NA	NA	NA	NA	0.38 (0.42)	0.38 (0.42)	-1.31 (0.30)	0.43 (0.35)	0.52 (0.42)	0.52 (0.42)	-1.02 (0.25)	0.53 (0.28)	NA	NA	NA	NA	**4.50 (<0.01)**	**4.51 (<0.01)**	**1.17 (<0.01)**	**2.33 (<0.01)**
**Hallucinations, auditory**																								

EMA	NA	NA	NA	NA	NA	NA	NA	NA	NA	NA	NA	NA	**1.24 (<0.01)**	**1.24 (<0.01)**	-0.79 (0.20)	0.72 (0.17)	NA	NA	NA	NA	**1.83 (<0.01)**	**1.83 (<0.01)**	-0.67 (0.16)	0.74 (0.16)
FAERS	NA	NA	NA	NA	NA	NA	NA	NA	0.35 (0.41)	0.35 (0.41)	-1.66 (0.34)	0.37 (0.39)	0.81 (0.28)	0.81 (0.28)	-0.71 (0.18)	0.68 (0.17)	NA	NA	NA	NA	**4.04 (<0.01)**	**4.04 (<0.01)**	**0.89 (<0.01)**	**1.95 (<0.01)**
**Psychotic disorder**																								
EMA	NA	NA	NA	NA	NA	NA	NA	NA	0.46 (0.57)	0.46 (0.57)	-2.08 (0.38)	0.40 (0.46)	0.67 (0.47)	0.67 (0.47)	-1.25 (0.28)	0.56 (0.31)	NA	NA	NA	NA	**3.40 (<0.01)**	**3.40 (<0.01)**	**0.21 (<0.01)**	**1.12 (<0.01)**
FAERS	0.31 (0.37)	0.31 (0.37)	-2.91 (0.44)	0.22 (0.45)	NA	NA	NA	NA	0.47 (0.42)	0.47 (0.42)	-1.11 (0.27)	0.50 (0.30)	0.81 (0.33)	0.81 (0.33)	-0.57 (0.14)	0.72 (0.14)	NA	NA	NA	NA	**3.13 (<0.01)**	**3.13 (<0.01)**	**0.82 (<0.01)**	**1.84 (<0.01)**
**Substance-induced psychotic disorder**																								
EMA	NA	NA	NA	NA	NA	NA	NA	NA	NA	NA	NA	NA	NA	NA	NA	NA	NA	NA	NA	NA	NA	NA	NA	NA
FAERS	NA	NA	NA	NA	NA	NA	NA	NA	NA	NA	NA	NA	0.81 (0.20)	0.81 (0.20)	-1.58 (0.34)	0.49 (0.31)	NA	NA	NA	NA	**4.21 (<0.01)**	**4.22 (<0.01)**	**0.02 (<0.01)**	**1.12 (<0.01)**

Table S3. Signal scores regarding adverse drug reactions other than abuse/dependence and withdrawal issues for selected opioid drugs (European Medicines Agency/EMA and the Food and Drug Administration-FDA Adverse Event Reporting System/FAERS datasets)

Boldface denotes signals based on FDR<0.05; Minimum number of events to compute signal statistics = 5 for all measures.

EMA: European Medicines Agency; EB05: 5% quantile of the posterior distribution of the empirical Bayesian geometric mean (estimated FDR); FAERS: Food and Drug Administration Adverse Event Reporting System; FDR: false discovery rate; IC025: 2.5% quantile of the posterior distribution of information component (estimated FDR); NA: not available (less than 5 events for this pair); PRR: proportional reporting ratio (estimated FDR); ROR: reporting odds ratios (estimated FDR).
