# Supplementary material for: Pharmacovigilance Signals of the Opioid Epidemic over 10 Years: Data Mining Methods in the Analysis of Pharmacovigilance Datasets Collecting Adverse Drug Reactions (ADRs) Reported to EudraVigilance (EV) and the FDA Adverse Event Reporting System (FAERS)
Source: Pharmaceuticals (Basel). 2022 May 27;15(6):675. doi: 10.3390/ph15060675 (PMC9231103; doi:10.3390/ph15060675)
Supplement: Supplementary file 1 [file pharmaceuticals-15-00675-s001.zip › TableS4_R1.pdf]

Table S4. Pharmacovigilance signals of opioid-related adverse drug reaction reports compared to benzodiazepine-related reports in the Food and Drug Administration Adverse Event Reporting System (FAERS)

Preferred term (PT)	PRR (FDR)	ROR (FDR)	IC (FDR)	EBGM (FDR)
**Misuse/abuse-related terms**				
Drug abuse	**1.03 (<0.01)**	**1.03 (<0.01)**	**-0.03 (0.01)**	**0.99 (0.01)**
Drug abuser	**1.15 (<0.01)**	**1.15 (<0.01)**	-0.42 (0.23)	0.83 (0.16)
Drug diversion	**5.86 (<0.01)**	**5.87 (<0.01)**	**0.65 (<0.01)**	**1.64 (<0.01)**
Drug use disorder	1.01 (0.11)	1.01 (0.11)	-0.17 (0.08)	0.92 (0.06)
Intentional product misuse	0.94 (0.44)	0.94 (0.44)	-0.13 (0.05)	0.93 (0.05)
Substance abuse	**3.88 (<0.01)**	**3.89 (<0.01)**	**0.57 (<0.01)**	**1.54 (<0.01)**
Substance use	0.79 (0.34)	0.79 (0.34)	-0.92 (0.37)	0.62 (0.32)
**Dependence-related terms**				
Dependence	**1.46 (<0.01)**	**1.46 (<0.01)**	**-0.03 (0.01)**	**1.04 (0.01)**
Drug dependence	**2.82 (<0.01)**	**2.83 (<0.01)**	**0.53 (<0.01)**	**1.47 (<0.01)**
Substance dependence	**12.82 (<0.01)**	**12.82 (<0.01)**	**0.57 (<0.01)**	**1.63 (<0.01)**
**Withdrawal-related terms**				
Drug withdrawal syndrome	**1.42 (<0.01)**	**1.42 (<0.01)**	**0.17 (<0.01)**	**1.15 (<0.01)**
**Overdose and off label use terms**				
Intentional overdose	0.38 (0.51)	0.38 (0.51)	-1.02 (0.38)	0.51 (0.37)
Off-label use	0.74 (0.51)	0.74 (0.51)	-0.29 (0.17)	0.82 (0.17)
Overdose	**1.41 (<0.01)**	**1.41 (<0.01)**	**0.19 (<0.01)**	**1.15 (<0.01)**
**Terms possibly associated with a misuse/abuse event**				
Acute psychosis	0.26 (0.50)	0.27 (0.50)	-1.99 (0.47)	0.29 (0.45)
Aggression	0.35 (0.51)	0.35 (0.51)	-1.12 (0.40)	0.47 (0.38)
Confusional state	0.77 (0.51)	0.76 (0.51)	-0.28 (0.16)	0.83 (0.16)
Delirium	**1.16 (<0.01)**	**1.16 (<0.01)**	**-0.01 (0.01)**	**1.02 (0.01)**
Euphoric mood	**2.48 (<0.01)**	**2.48 (<0.01)**	**0.37 (<0.01)**	**1.35 (<0.01)**
Feeling of relaxation	0.32 (0.47)	0.32 (0.47)	-2.24 (0.48)	0.28 (0.45)
Hallucination, auditory	0.24 (0.51)	0.24 (0.51)	-1.67 (0.45)	0.33 (0.44)
Hallucination, visual	0.62 (0.51)	0.62 (0.51)	-0.61 (0.30)	0.68 (0.28)
Psychotic disorder	0.24 (0.51)	0.24 (0.51)	-1.62 (0.45)	0.34 (0.43)
Substance-induced psychotic disorder	0.73 (0.42)	0.73 (0.42)	-0.82 (0.35)	0.64 (0.31)

Boldface denotes signals based on FDR<0.05; minimum number of events to compute signal statistics is five for all measures.

EBGM: empirical Bayesian geometric mean; FAERS: Food and Drug Administration Adverse Event Reporting System; FDR: false discovery rate; IC: information component; NA: not available (less than five events for this pair); PRR: proportional reporting ratio; ROR: reporting odds ratio.

Opioids included in the analysis: codeine, dihydrocodeine, fentanyl, oxycodone, pentazocine, tramadol.

Benzodiazepines included in the analysis: diazepam, alprazolam, clonazepam, lorazepam, delorazepam, bromazepam, flurazepam, triazolam.
