# Supplementary material for: Pharmacovigilance Signals of the Opioid Epidemic over 10 Years: Data Mining Methods in the Analysis of Pharmacovigilance Datasets Collecting Adverse Drug Reactions (ADRs) Reported to EudraVigilance (EV) and the FDA Adverse Event Reporting System (FAERS)
Source: Pharmaceuticals (Basel). 2022 May 27;15(6):675. doi: 10.3390/ph15060675 (PMC9231103; doi:10.3390/ph15060675)
Supplement: Supplementary file 1 [file pharmaceuticals-15-00675-s001.zip › TableS5_opioids_NPS_R1.pdf]

EV local number	Opioid drug *	Concomitant drugs *	*Traditional* illicit drugs *	NPS *	Country of occurrence	ADR recorded	Outcome	Notes
**EMA**								
11254486	Tramadol	• Loperamide	Not recorded	• Mitragynine	USA	• Drug abuse<br>• Accidental drug overdose<br>• Cardiomegaly<br>• Possible drug interaction	Fatal	
12734320	Tramadol	• Aripiprazole<br>• Haloperidol<br>• Hydroxyzine	• 3,4-methylenedioxymethamphetamine<br>• Cocaine	• 4-methylethcathinone (injected)<br>• Mephedrone	France	• Drug use disorder<br>• Delirium<br>• Multiple drug abuse<br>• Tachycardia<br>• Intentional drug misuse<br>• Drug addiction	Prolonged hospitalization	
12859189	Tramadol (tablet)	• Loperamide	Not recorded	• Mitragynine	USA	• Product used for unknown indication<br>• Accidental overdose<br>• Drug abuse<br>• Drug interaction<br>• Toxicity to various agents<br>• Cardiomegaly	Fatal	Collapsed while playing basketball
6324477	Tramadol	Not recorded	Not recorded	• Methylenedioxypyrovalerone	USA	• Drug abuse<br>• Unknown cause of death<br>• Cardiopulmonary arrest	Fatal	
**FAERS**								
7389476	Codeine	• Doxylamine	• Heroin	• Mephedrone	NA	• Drug abuse<br>• Toxicity to various agents	Fatal	Male
7945914	Codeine	• Morphine<br>• Doxylamine	• Heroin	• Mephedrone	NA	• Drug abuse<br>• Toxicity to various agents	Fatal	Male
14765776	Codeine	• Lorazepam	Not recorded	• Methylenedioxypyrovalerone	NA	• Product used for unknown indication<br>• Disturbance in attention<br>• Dysarthria<br>• Daydreaming<br>• Somnolence<br>• Accident	Not recorded	Male

						• Toxicity to various agents<br>• Gait Disturbance		
14769546	Codeine	• Morphine<br>• Diazepam	• Cocaine	• Methylenedioxypyrovalerone	NA	• Product used for unknown indication<br>• Vestibular disorder<br>• Thinking Abnormal<br>• Dysarthria<br>• Slow response to stimuli<br>• Disturbance in attention<br>• Fine motor skill dysfunction<br>• Logorrhoea<br>• Toxicity to various agents<br>• Memory impairment<br>• Mood swings	Not recorded	Male
14771196	Codeine	• Diazepam<br>• Opipramol<br>• Ibuprofen<br>• Lorazepam<br>• Levomethadone	Not recorded	• Methylenedioxypyrovalerone	NA	• Product used for unknown indication<br>• Toxicity to various agents<br>• Patient uncooperative<br>• Nervous system disorder<br>• Aggression<br>• Behaviour disorder	Not recorded	Female
8723789	Fentanyl	Not recorded	Not recorded	• Methylenedioxypyrovalerone	NA	• Pneumonia aspiration<br>• Bacteraemia<br>• Aggression<br>• Cognitive disorder<br>• Blood pressure diastolic decreased<br>• Serotonin syndrome<br>• Pneumothorax<br>• Hallucination<br>• Confusional state<br>• Myoclonus<br>• White blood cell count increased<br>• Hypertonia	Hospitalized	Female on analgesic therapy
8726306	Fentanyl	Not recorded	Not recorded	• Methylenedioxypyrovalerone	NA	• Condition aggravated<br>• Bacteraemia<br>• Hyperreflexia<br>• Hypertonia<br>• Agitation	Hospitalized	Female on analgesic therapy

						• Clonus<br>• Pneumonia aspiration<br>• Aggression<br>• Serotonin syndrome<br>• Pneumothorax		
12639421	Fentanyl	• Alprazolam<br>• Clonazepam	Not recorded	• Mitragynine	NA	• Urinary retention<br>• Left ventricular hypertrophy<br>• Respiratory arrest<br>• Accidental death<br>• Drug screen positive<br>• Pulmonary congestion<br>• Toxicity to various agents<br>• Oedema peripheral	Fatal	Male
14561508	Fentanyl	• Quetiapine<br>• Doxepin<br>• Alprazolam<br>• Alcohol	Not recorded	• Methylenedioxypyrovaler one	NA	• Product used for unknown indication<br>• Crime<br>• Somnolence<br>• Toxicity to various agents<br>• Aggression	Not recorded	Male
14765886	Fentanyl	• Methadone<br>• Lorazepam<br>• Pseudoephedrine<br>• Pregabalin<br>• Methamphetamine<br>• Trimipramine<br>• Morphine	Not recorded	• Methylenedioxypyrovaler one	NA	• Product used for unknown indication<br>• Toxicity to various agents	Not recorded	Male
4179421	Oxycodon e	• Propoxyphene<br>• Naproxen	Not recorded	• Phenethylamine	NA	• Accidental overdose	Fatal	Male
9823195	Oxycodon e	• Hydromorphone hydrochloride	Not recorded	• Mitragynine	NA	• Substance abuse<br>• Drug withdrawal syndrome<br>• Pain	Not recorded	Male
10690199	Oxycodon e	Not recorded	Not recorded	• Mephedrone	NA	• Product used for unknown indication<br>• Drug diversion<br>• Unresponsive to stimuli<br>• Intentional overdose<br>• Suicide attempt<br>• Loss of consciousness	Hospitalized	Male

10737377	Oxycodone	• Lorazepam<br>• Meprobamate	Not recorded	• Alpha-Pyrrolidinopropiophenone	NA	• Completed suicide<br>• Respiratory arrest<br>• Cardiac arrest	Fatal	Male
10772317	Oxycodone	• Lorazepam<br>Meprobamate	Not recorded	• Alpha-Pyrrolidinopropiophenone	NA	• Product used for unknown indication<br>• Toxicity to various agents<br>• Completed suicide<br>• Respiratory arrest<br>• Cardiac arrest	Fatal	Male
13669186	Oxycodone	• Dronabinol	• Amphetamine	• Flubromazolam<br>• 3-Methoxyphencyclidine<br>• 4-Methoxyphencyclidine	NA	• Product used for unknown indication<br>• Brain oedema<br>• Toxicity to various agents<br>• Pneumonia<br>• Myocardial oedema<br>• Heart injury<br>• Visceral congestion<br>• Visceral oedema<br>• Pulmonary oedema	Fatal	Male
8083892	Tramadol	• Venlafaxine<br>• Trimeprazine tartrate<br>• Mirtazapine<br>• Diazepam<br>• Buprenorphine	Not recorded	• Mitragynine	NA	• Product used for unknown indication<br>• Pulmonary oedema<br>• Accidental Death<br>• Brain oedema<br>• Overdose	Fatal	Male
8121536	Tramadol	• Venlafaxine<br>• Mirtazapine<br>• Diazepam<br>• Buprenorphine	Not recorded	• Mitragynine	NA	• Toxicity to various agents<br>• Pulmonary oedema<br>• Loss of consciousness<br>• Cardiac arrest<br>• Brain oedema	Fatal	Male
8121551	Tramadol	• Pregabalin<br>• Fluoxetine<br>• Olanzapine<br>• Diazepam	Not recorded	• Mitragynine	NA	• Toxicity to various agents<br>• Hepatic steatosis<br>• Cardiac arrest<br>• Loss of consciousness<br>• Pulmonary congestion	Fatal	Female
8121559	Tramadol	• Zopiclone<br>• Venlafaxine	Not recorded	• Mitragynine	NA	• Toxicity to various agents<br>• Respiratory depression<br>• Loss of consciousness	Fatal	Female

						• Pulmonary congestion<br>• Cardiac arrest<br>• Accidental overdose		
8121566	Tramadol	• Trimeprazine artrate	Not recorded	• Mitragynine	NA	• Toxicity to various agents<br>• Pulmonary oedema	Fatal	Male
8124388	Tramadol	• Cannabis sativa extract<br>• Alprazolam	• Amphetamine	• Mitragynine	NA	• Accidental overdose<br>• Toxicity to various agents	Fatal	Male
8124494	Tramadol	• Alprazolam<br>• Alcohol	Not recorded	• Mitragynine	NA	• Toxicity to various agents<br>• Pulmonary congestion	Fatal	Male
8782890	Tramadol	• Cannabis sativa extract<br>• Alprazolam<br>• Citalopram	Not recorded	• Mitragynine	NA	• Toxicity to various agents<br>• Pulmonary oedema<br>• Drug level increased<br>• Respiratory depression<br>• Overdose<br>• Brain oedema	Fatal	Male
8132531	Tramadol	Alcohol	Not recorded	• Mitragynine	NA	• Pain<br>• Rib fracture<br>• Tachycardia<br>• Blood ethanol increased<br>• Death<br>• Cardiac arrest<br>• Blood glucose increased<br>• Toxicity to various agents<br>• Arrhythmia<br>• Drug ineffective<br>• Coronary artery stenosis	Fatal	Male
9013356	Tramadol	Not recorded	Not recorded	• Methylenedioxypyrovalerone	NA	• Product used for unknown indication	Fatal	Male
9023990	Tramadol	Not recorded	Not recorded	• Methylenedioxypyrovalerone	NA	• Toxicity to various agents<br>• Drug abuse<br>• Product used for unknown indication	Fatal	Male
9103060	Tramadol	Not recorded	Not recorded	• Methylenedioxypyrovalerone	NA	• Cardio-respiratory arrest<br>• Poisoning death<br>• Drug abuse<br>• Respiratory arrest<br>• Cardiac arrest	Fatal	Male

9146885	Tramadol	Not recorded	Not recorded	• Methylenedioxypyrovalerone	NA	• Drug abuse<br>• Respiratory arrest<br>• Cardiac arrest	Fatal	Male
9147109	Tramadol	Not recorded	Not recorded	• Methylenedioxypyrovalerone	NA	• Product used for unknown indication	Fatal	Male
12850962	Tramadol	• Loperamide	Not recorded	• Mitragynine	NA	• Drug abuse<br>• Product used for unknown indication<br>• Respiratory arrest<br>• Cardiac arrest	Fatal	Male
13066343	Tramadol	• Loperamide	Not recorded	• Mitragynine	NA	• Drug abuse<br>• Respiratory arrest<br>• Cardiac arrest<br>• Product used for unknown indication<br>• Drug interaction<br>• Accidental overdose<br>• Cardiomegaly	Fatal	Male
14331361	Tramadol	Not recorded	Not recorded	• 3-Methoxyphencyclidine	NA	• Product used for unknown indication<br>• Depressed level of consciousness<br>• Respiratory depression<br>• Overdose<br>• Tachycardia	Hospitalized	Male
14770087	Tramadol	• Doxepin<br>• Diazepam	Not recorded	• Methylenedioxypyrovalerone	NA	• Product used for unknown indication<br>• Aggression<br>• Logorrhoea<br>• Dysarthria<br>• Restlessness<br>• Toxicity to various agents<br>• Abnormal behaviour<br>• Gait disturbance	Not recorded	Male

Table S5. Description of cases involving opioids and new psychoactive substances (NPS) recorded in both the European Medicines Agency (EMA) and the Food and Drug Administration Adverse Event Reporting System (FAERS) databases

*Dosages and routes of administration were not recorded

ADR: adverse drug reaction; EV: EudraVigilance; NA: not available; NPS: new psychoactive substance; USA: United States of America
